# Efficient production of salvianic acid A from l-dihydroxyphenylalanine through a tri-enzyme cascade

**DOI:** 10.1186/s40643-023-00649-0

**Published:** 2023-05-01

**Authors:** Jiahui Yang, Wanqing Wei, Changzheng Gao, Wei Song, Cong Gao, Xiulai Chen, Jia Liu, Liang Guo, Liming Liu, Jing Wu

**Affiliations:** 1 School of Life Science and Health Engineering, Jiangnan University, Wuxi, 214122 China; 2 State Key Laboratory of Food Science and Technology, Jiangnan University, Wuxi, 214122 China; 3 Department of Cardiology, Affiliated Hospital of Jiangnan University, Wuxi, 214122 China

**Keywords:** Salvianic acid A, Tri-enzyme cascade, Whole-cell biotransformation, Protein engineering

## Abstract

Salvianic acid A (SAA), used for treating cardiovascular and cerebrovascular diseases, possesses several pharmacological properties. However, the current methods for the enzymatic synthesis of SAA show low efficiency. Here, we constructed a three-enzyme cascade pathway in *Escherichia coli* BL21 (DE3) to produce SAA from l-dihydroxyphenylalanine (L-DOPA). The phenylpyruvate reductase (*La*PPR) from *Lactobacillus sp. CGMCC 9967* is a rate-limiting enzyme in this process. Therefore, we employed a mechanism-guided protein engineering strategy to shorten the transfer distances of protons and hydrides, generating an optimal *La*PPR mutant, *La*PPR^Mu2^ (H89M/H143D/P256C), with a 2.8-fold increase in specific activity and 9.3-time increase in *k*_cat_/*K*_m_ value compared to that of the wild type. Introduction of the mutant *La*PPR^Mu2^ into the cascade pathway and the optimization of enzyme levels and transformation conditions allowed the obtainment of the highest SAA titer (82.6 g L^−1^) ever reported in vivo, good conversion rate (91.3%), excellent ee value (99%) and the highest productivity (6.9 g L^−1^ h^−1^) from 90 g L^−1^ L-DOPA in 12 h*.* This successful strategy provides a potential new method for the industrial production of SAA.

**Graphical Abstract:**

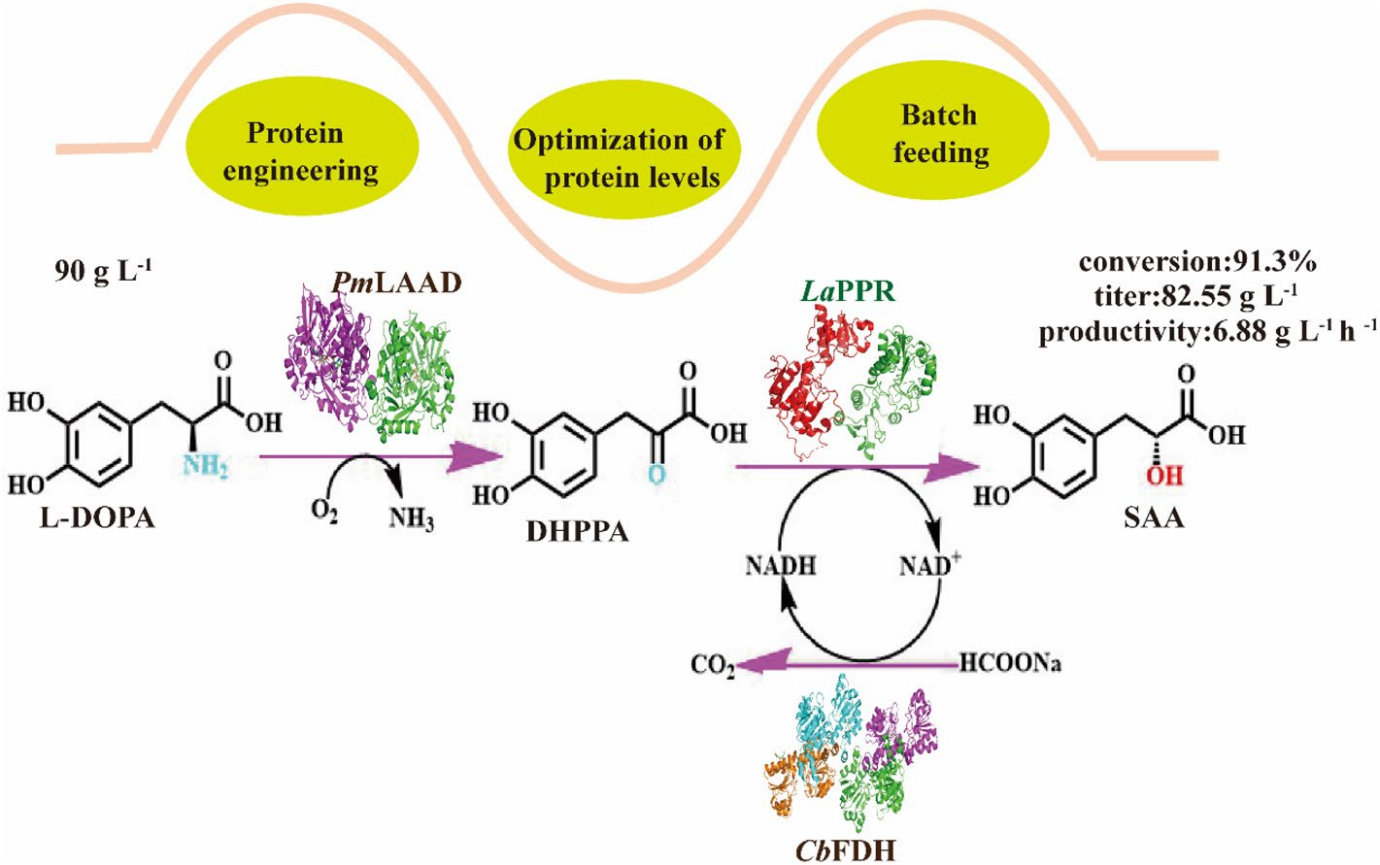

**Supplementary Information:**

The online version contains supplementary material available at 10.1186/s40643-023-00649-0.

## Introduction

**Fig. 1:**
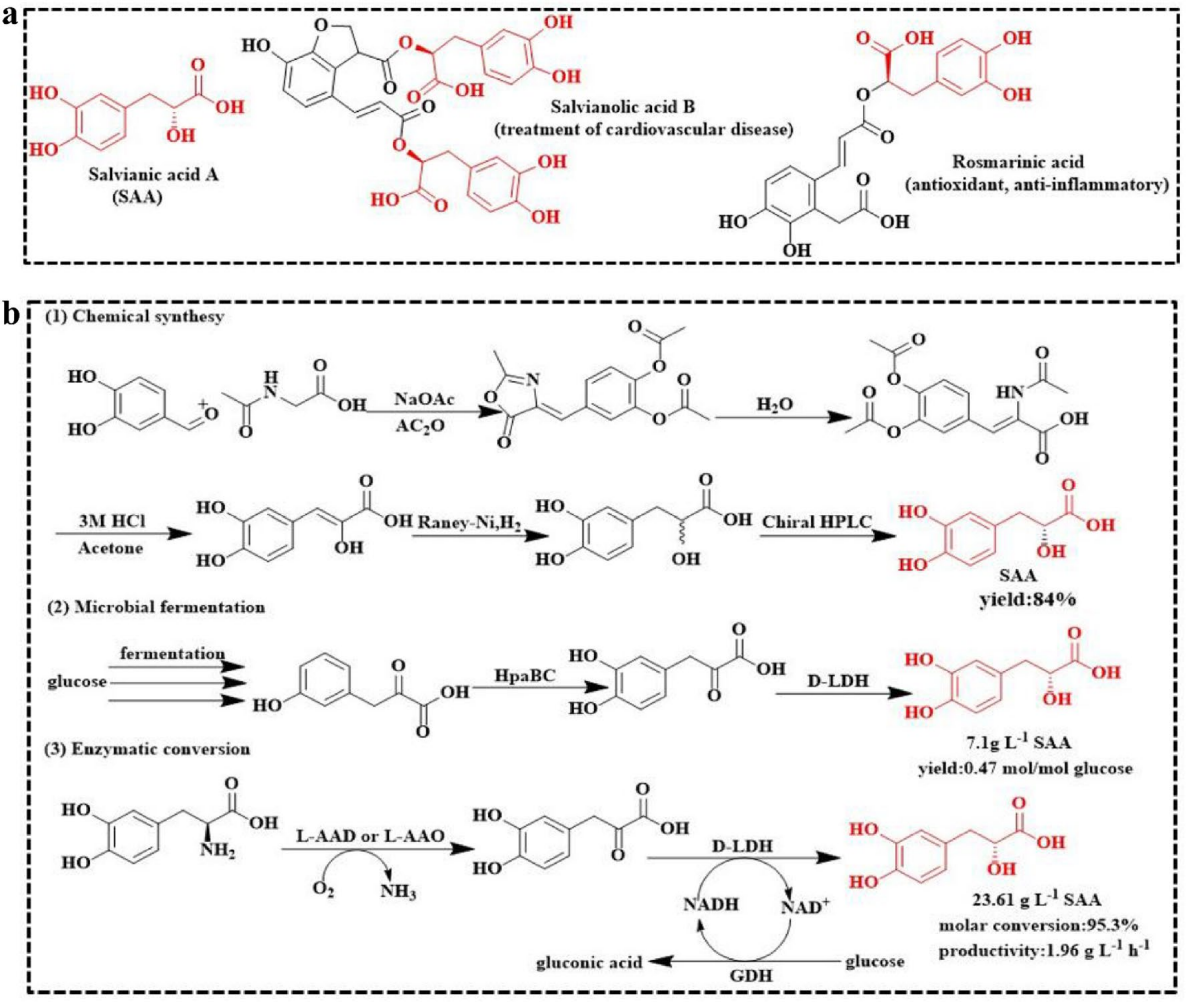
Application and synthetic route of SAA. **a** The derivative products of SAA. **b** Chemical synthesis, microbial fermentation and enzymatic conversion of SAA at present

Salvianic acid A (SAA) was originally extracted from the roots of the traditional Chinese plant *Salvia miltiorrhiza* (Mahalakshmi et al. 2021) and is mainly used in treating cardiovascular and cerebrovascular diseases (Huo et al. 2017; Li et al. 2018; Zhang et al. 2019). SAA also exhibits anti-fibrosis (Cao et al. 2019), anti-tumor (Kumar et al. 2020) and anti-inflammation properties (Sun et al. 2020). Moreover, SAA can be used as substrate for the synthesis of several drugs (Cui et al. 2014), such as rosmarinic acid (Yin et al. 2015), salvianolic acid B (S P Wang et al. 2008) (Fig. 1a).

There are two main methods for synthesizing SAA: chemical synthesis and biosynthesis (Fig. 1b). In the industrial production of sodium acetate, acetylglycine, and 3,4-dihydroxybenzaldehyde as raw materials, racemic SAA is synthesized by condensation, ring-opening, hydrolysis, and hydrogenation, and SAA obtained through chiral separation (Bai et al. 2013). However, this method shows poor stereoselectivity, several laborious steps, harsh reaction conditions (high temperature and pressure), and the potential to cause environmental pollution. SAA biosynthesis has attracted increasing attention because of its high stereoselectivity, mild process conditions, and the potential to reduce pressure on the environment and resources (L Zhou et al. 2017). SAA biosynthesis involves microbial fermentation and enzymatic conversion. For microbial fermentation, 4-hydroxyphenylpyruvate resulting from the fermentation of 13.7 g/L glucose in *E. coli*, is first hydroxylated by the hydroxylase complex (HpaBC) and then reduced by D-lactate dehydrogenase (D-LDH) to produce SAA (with a titer of 7.1 g/L and a yield of 0.47 mol/mol glucose) (Yao et al. 2013). However, this method is limited by a long fermentation time (70 h), lower SAA titer (7.1 g/L), and SAA was excessive oxidized. As for enzymatic conversion (Hu et al. 2022; Xiong et al. 2019a, b, c), l-dihydroxyphenylalanine (L-DOPA) is converted to 3,4-dihydroxyphenylpyruvic acid (DHPPA) by L-amino acid deaminase (L-AAD) (Motta et al. 2016) or L-amino acid oxidase (L-AAO) (Du and Clemetson 2002) and subsequently reduced by D-LDH to SAA. Concomitantly, glucose dehydrogenase (GDH) coupling occurs for NADH regeneration (France et al. 2016; Guo et al. 2016). Although this method presents the highest conversion rate (95.3%) of SAA, its industrial application is limited by a low titer (23.6 g/L) and productivity (2.0 g L^−1^ h^−1^), caused by insufficient D-LDH enzyme activity (Xiong et al. 2019a, b, c). Therefore, genome mining or rational protein engineering of highly efficient α-ketone acid reductases might be pivotal for achieving large-scale efficient production of SAA.

**Fig. 2:**
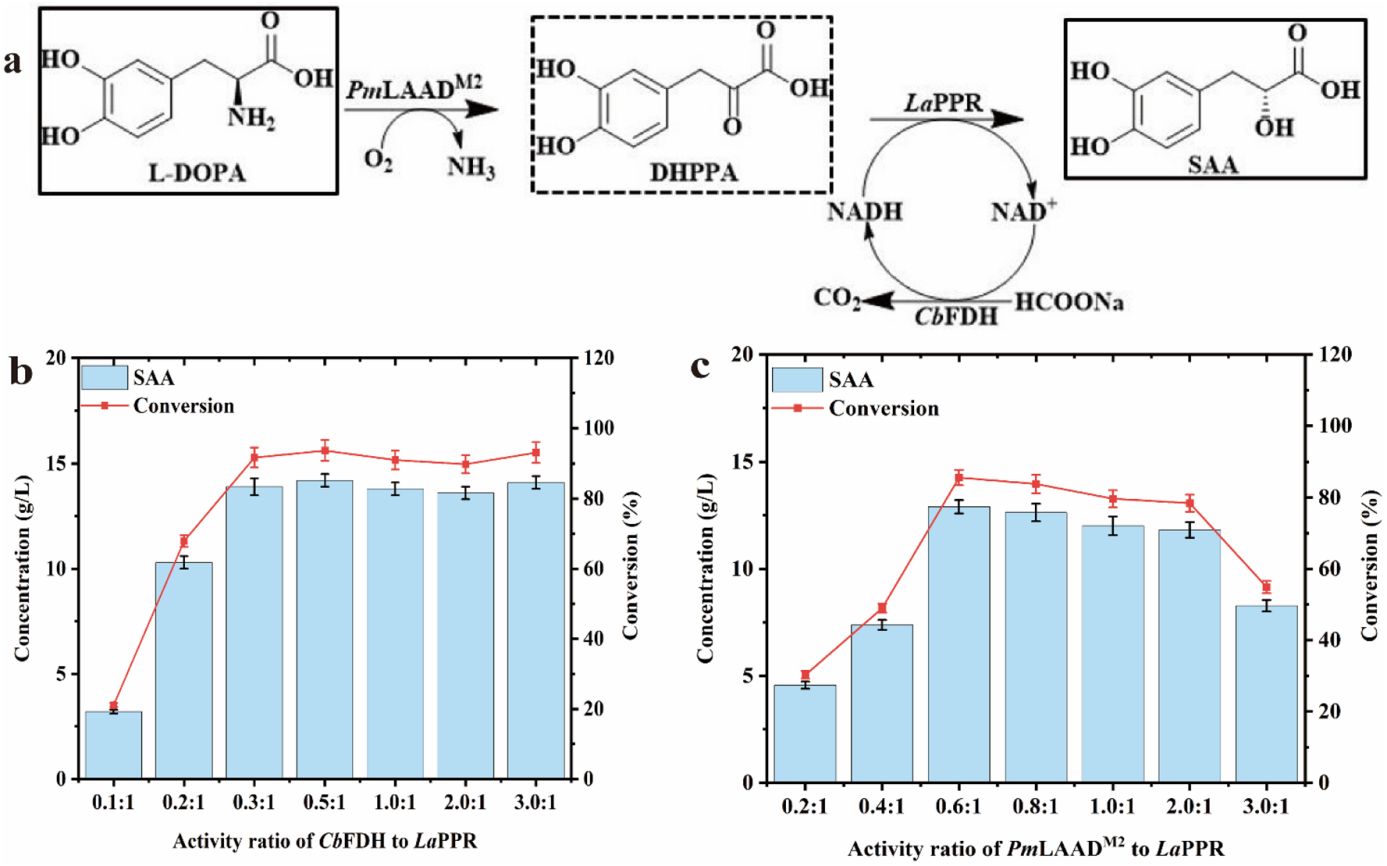
Design and construct a tri-enzymatic cascade pathway to synthesize SAA from L-DOPA. **a** Designed SAA biosynthesis pathway by a tri-enzyme cascade pathway containing *Pm*LAAD^**M2**^, *La*PPR and *Cb*FDH; **b** effect of different activity ratios (0.1:1–3:1) of *Cb*FDH/*La*PPR on SAA production, with 15 g L^−1^ DHPPA, 16 g L^−1^ sodium formate in Tris–HCl buffer (50 mM, pH 7.0, containing 5 mM NAD^+^) in 30 ℃ for 12 h. **c** When the enzyme activity ratio of *Cb*FDH: *La*PPR was maintained at 0.3:1, effect of different activity ratios (0.2:1–3:1) of *Pm*LAAD^**M2**^/*La*PPR on SAA production, with 15 g L^−1^ L-DOPA, 16 g L^−1^ sodium formate, in Tris–HCl buffer (50 mM, pH 7.0, containing 5 mM NAD^+^, 5 mM FAD) in 30 ℃ for 12 h. The data represent mean ± s.d., as determined from three independent experiments

Here, we constructed a tri-enzyme cascade pathway capable of converting L-DOPA to SAA in *Escherichia coli* BL21 (DE3) (Fig. 2a). It contained L-amino acid deaminase mutant from *Proteus mirabilis* (*Pm*LAAD^M2^), phenylpyruvate reductase from *Lactobacillus* sp.CGMCC 9967 (*La*PPR), and formate dehydrogenase from *Candida boidinii* (*Cb*FDH). The *La*PPR was determined to be the rate-limiting enzyme in SAA synthesis. To improve the activity of *La*PPR, we generated an optimal mutant *La*PPR^**Mu2**^ (H89M/H143D/P256C), through mechanism-guided protein engineering, with a 2.8-fold increase in specific activity and 9.3-time increase in *k*_cat_/*K*_m_ value compared to the wild type. Through integration of the best triple mutant *La*PPR^**Mu2**^ into the cascade pathway, optimization of the plasmid copy number, ribosome-binding site (RBS) sequence, and transformation conditions, we achieved the synthesis of 82.6 g L^−1^ SAA (the highest so far) from 90 g L^−1^ L-DOPA (batch feeding) within 12 h; with a molar conversion rate of 91.3%, ee value > 99%, with the highest productivity reported so far (6.88 g L^−1^ h^−1^), to the best of our knowledge.

## Materials and methods

### Materials

*Escherichia coli* JM109 were used for plasmid amplifications and *E. coli* BL21 (DE3) (Carlsbad, CA, U.S.A.) for recombinant enzyme productions. Gene expression was achieved by cloning the desired gene(s) into a set of plasmids pET28a ( +), pETDuet-1, pRSFDuet-1 and pCDFDuet-1 (Novagen, Darmstadt, Germany). Various antibiotics (ampicillin 100 μg/mL, kanamycin 50 μg/mL, and streptomycin 50 μg/mL) were added to the media containing *E. coli* strains bearing plasmid. L-DOPA and SAA were purchased from Aladdin (Shanghai, China). 3,4-Dihydroxyphenylpyruvic acid (DHPPA) was obtained from Bide pharmatech (Shanghai, China). PrimeSTAR HS DNA Polymerase, T4 DNA ligase, and all restriction enzymes were from TaKaRa (Dalian, China). All other reagents and chemicals (analytical grade) were purchased from Sinopharm (Beijing, China).

### Construction of the co-expressed strains

Main primers used to construct co-expressed strains are summarized in Additional file 1: Table S1. The genes of *Pm*LAAD (GenBank: MG746627.1), *La*PPR (GenBank: KP735960.1) and *Cb*FDH (GenBank: X81129.1) were all derived from the host bacterium genome, which was obtained by polymerase chain reaction (PCR) using the genomes of the *Proteus mirabilis*, *Lactobacillus* sp.CGMCC 9967 and *Candida boidinii* as templates. According to our previous results (Wu et al. 2021), the *Pm*LAAD^**M2**^ (H295S/V437S) gene was obtained from the mutation of *Pm*LAAD gene. The *La*PPR gene was inserted between BamHI and HindIII restriction sites that after the first T7 promoter, the *Pm*LAAD^**M2**^ gene was inserted between NdeI and KpnI restriction sites that after the second T7 promoter, and the *Cb*FDH gene was linked to *Pm*LAAD^**M2**^ by using an RBS sequence, insert between KpnI and XhoI restriction sites.

### Computational experiments

#### AlphaFold prediction

The energy-minimized structural model of *La*PPR^**Mu2**^ was downloaded from AlphaFold (Jumper et al. 2021). The model quality was evaluated by Verfy-3D and the Ramachandran plot by SAVES 6.0 (https://saves.mbi.ucla.edu/). The Verify 3D analysis showed that 94.3% amino acid 3D-1D score was above 0.2 (At least 80% of the amino acids have scored ≥ 0.2 in the 3D/1D profile.) The Ramachandran plot showed that 94.7% of the total number of residues were in the favored region, indicating reliability of the model.

#### Initial structural preparation

The initial structure of *La*PPR was based on the crystal structure (PDB ID: 8HPG) and the *La*PPR–NADH binary complex by overlapping with the complex crystal structure of phosphoglycerate dehydrogenase-NADH (PDB ID: 2EKL) (Singh et al. 2014) was constructed. The protonation states of charged residues were determined at constant pH 7.0 based on pKa calculations via the H +  + (Gordon et al. 2005) and the local hydrogen bonding network. The residues His79, 158, 165 and 182 were assigned as HID, His78, 203, 272 were HIP and the rest were HIE. Asp and Glu residues were deprotonated, while Lys and Arg were protonated.

#### Molecular docking

The 3D structure of DHPPA molecules were downloaded from the PubChem (https://www.ncbi.nlm.nih.gov). DHPPA was fully optimized at the B3LYP/6-31G(d) level by Gaussian 16 package (Frisch et al. 2016) and then docked into the active site of the *La*PPR–NADH binary complex. Molecular docking was performed using the Lamarckian genetic algorithm local search method in AutoDock 4.2 and AutoDockTools-1.5.6. A docking approach was employed for rigid receptor conformation. In total, 100 independent docking runs were performed and reasonable conformations were selected as the binding conformations for *La*PPR–NADH–DHPPA molecular dynamics (MD). The analysis of the structures was conducted on PyMOL 2.3 (Schrodinger (SDGR)), and the optimal docking conformation was selected in the principle of the binding energy and possible catalytically reactive conformation.

#### Molecular dynamics simulation

All molecular dynamics (MD) simulations were performed by the Amber 16 package (Case. et al. 2016). The pre-equilibrated *La*PPR and NADH structures, as well as the possible catalytically reactive binding modes of DHPPA were used as the starting conformations for MD simulations of the protein–ligand complexes. A representative pre-equilibrated structure of *La*PPR–NADH–DHPPA was used as template for creating the starting coordinate for *La*PPR^**Mu2**^–NADH–DHPPA with the modification of the three mutated residues (H89M/H143D/P256C). The partial charges of NADH and DHPPA were fitted with HF/6-31G(d) calculations and the restrained electrostatic potential (RESP) protocol (Araz Jakalian et al. 2000; A. Jakalian et al. 2002) was implemented using the Antechamber module of the Amber 16 package (Jorgensen et al. 1983). The prepared protein was neutralized by adding Na^+^ ions and solvated into a truncated octahedron TIP3P (Jorgensen et al. 1983) water box with a 10 Å buffer distance on each side. The resulting system contained 52,158 *La*PPR–NADH–DHPPA and 52,116 *La*PPR^**Mu2**^–NADH–DHPPA atoms. Next, each system was equilibrated with a series of minimizations interspersed by short MD simulations, during which restraints on the protein backbone heavy atoms were gradually released (with force constants of 10, 2, 0.1, and 0 kcal/(mol·Å^2^)) and heated slowly from 0 to 303 K for 50 ps, in which we applied a 5 kcal/(mol·Å^2^) restraint on the protein backbone heavy atoms. Finally, extensive MD simulations of 110 ns were performed at constant temperature and pressure, in which the heavy atoms of the protein backbone were restrained with a force constant of 1 kcal/(mol·Å^2^) during the first 10 ns, after which the remaining 100 ns were unrestrained. The pressure was maintained at 1 atm and coupled with isotropic position-scaling. The temperature was stabilized at 303 K using the Berendsen thermostat method (Berendsen et al. 1984). Long-range electrostatic interactions were treated using the particle mesh Ewald (PME) method (Darden et al. 1993). A 12 Å cutoff was applied to both PME and van der Waals (vdW) interactions. A time step of 2 fs was employed along with the SHAKE algorithm (Sharma et al. 2020) for hydrogens, and a periodic boundary condition. Atomic positions were stored every 2 ps for further analysis. Each system was checked for stability (structure, energy, and temperature fluctuations) and convergence (root mean square deviations (RMSD) of the structures).

### Construction and screening of mutagenesis libraries

The mutant library was constructed through the PCR of the whole-plasmids using KOD-Plus-Neo (TOYOBO, Osaka, Japan), employing plasmid pET28a-*La*PPR as the template. The primers used for mutant construction are listed in Additional file 1: Table S1. The resulting PCR products were digested with DpnI to remove the template plasmid, after which, 10 μL of the digested products was transformed in *E. coli* BL21 (DE3) cells for subsequent screening or DNA sequencing (Suzhou, China). The single colonies in culture dishes were randomly picked and cultured into 500 μL LB medium with 50 μg mL^−1^ kanamycin in 96-well plate and shaken at 37 °C for 8–12 h. Then, they were 1:10 diluted into 500 μL fresh TBA medium in new 96-well plate (containing 4 g/L lactose). After shaking at 37 °C for 3 h (for cell growth), the temperature was decreased to 25 °C for 14 h (for protein expression). Then, the cells were harvested by centrifugation at 3700 ×*g* at 4 °C for 15 min. To screen the positive mutants from a 96-well plate, the supernatants were prepared and screened for activity using the monitorization of NADH consumption at 30 °C in a reaction system of Tris–HCl buffer (50 mM, pH 7.0), containing 160 µL crude enzyme extract, 20 µL substrate (100 mM DHPPA), and 20 µL of NADH (10 mM). Mutants with twofold higher activity toward DHPPA than that of the wild-type enzyme were selected for further experiments.

### Protein expression and purification of *La*PPR

The recombinant *E. coli* strains containing *La*PPR was cultured in LB medium containing kanamycin (50 μg/ml) at 37 °C and 180 rpm. When the optical density (OD_600_) of the culture reached 0.6–0.8, 0.4 mM IPTG (final concentration) was added to induce enzyme expression and incubated at 25 °C for 14 h. The cells were collected by centrifugation (4 ℃, 6000 ×*g*, 10 min), and resuspended in buffer A (150 mM NaCl, 20 mM imidazole, 25 mM Tris–HCl buffer, pH 7.4). Cell suspensions were lysed through sonication and centrifuged at 14,000 × g for 0.5 h. The subsequent experiments were performed on an ÄKTA pure system (GE Healthcare) with a HisTrap HP column (5 ml, GE Healthcare). The protein was then concentrated for activity assay or further purification using one more exclusion column (Superdex 200 16/600, GE Healthcare) and an elution buffer (25 mM Tris–HCl, pH 7.4, 150 mM NaCl) for crystallization. The protein concentration of the purified enzyme was determined using a NanoDrop 2000c spectrophotometer at 280 nm (Thermo Scientific, Waltham, MA, USA), considering the extinction coefficients calculated with the ExPASy ProtParam Tool. Purification procedures were conducted following standard protocols and manufacturer’s information, most reactions were conducted at 4 °C.

### Protein crystallization and structure determination

All initial crystallization conditions were screened by the sitting drop vapor diffusion method using the Hampton Research Crystal Screen Kits. The crystal for data collection of *La*PPR wild-type enzyme was obtained after 3 days at 20 °C in 96-well plates, using 0.8 μL of protein solution (28 mg/mL) and an equal volume of reservoir solution (0.1 M Citric acid pH 3.5, 14%*w*/*v* PEG 1000 and 0.01 M B-Nicotinamide adenine dinucleotide). All the crystals were flash-frozen in liquid nitrogen. Data were collected at beamlines BL19U1 and BL18U1 at the Shanghai Synchrotron Radiation Facility (SSRF, Shanghai, China) and the crystal diffraction data file (sca file and log file) is output through the HKL-3000 package. CCP4 software is used to convert sca files into protein electronic density files (mtz files). The Mattews_coef module in CCP4 software package is used to calculate the number of molecules in each cell. Using the crystal structure of phosphoglycerate dehydrogenase from *Lactobacillus plantarum* as template (PDB: 3EVT), the crystal phase of *La*PPR is determined by molecular replacement of the protein crystal template using Phase_MR module in CCP4 software package, and the protein pdb file is output at the same time. The obtained electron cloud density file and structure file need to undergo iterative cycles of model building and refinement through Phenix and WinCoot software packages to meet the qualified crystal standards. The data collection and refinement statistics of the *La*PPR crystal structure are listed in Additional file 1: Table S2 and had been deposited in the PDB under the accession code 8HPG. Structural figures were prepared using PyMOL v2.3.3 (Schrödinger, LLC, USA).

### Enzymatic activity assay

Enzymatic activity was analyzed as previously described (Song et al. 2018). *Pm*LAAD (EC: 1.4.3.2) activity on L-DOPA was assayed by coupling DHPPA formation. The reaction system (50 mM Tris–HCl buffer, pH 7.0, 30 °C) contained 20 g L^−1^ L-DOPA. One unit of *Pm*LAAD activity (U) was defined as the amount of enzyme necessary for production of 1 µmol of DHPPA in 1 min. *La*PPR (EC: 1.1.1.49) activity on DHPPA was assayed by coupling NADH consumption. The reaction mixture contained 50 mM DHPPA, and 5 mM NADH. One unit of *La*PPR activity (U) was defined as the amount of enzyme consuming 1 µmol of NADH in 1 min. *Cb*FDH (EC: 1.2.1.2) activity toward sodium formate was assayed by coupling NADH formation. The reaction mixture comprised 50 mM sodium formate, 5 mM NAD^+^. One unit of *Cb*FDH activity (U) was defined as the amount of enzyme necessary for production of 1 µmol of NADH in 1 min. The activity of the recombinant strains expressing a single enzyme was measured using purified enzymes, while the activity of the co-expressed strains was measured using wet whole-cell catalysts.

### Determination of kinetic parameters

Kinetic parameters for the reduction of DHPPA were determined at 30 °C in Tris–HCl buffer (50 mM, pH 7.0) contained 2 mM NADH, with DHPPA concentration varied from 1 to 50 mM. *La*PPR was added to initiate the reaction, and the absorbance was monitored at 340 nm. All experiments were conducted in triplicates. *K*_*m*_ and *k*_*cat*_ were calculated by nonlinear fitting using Origin software.

### HPLC analysis of DHPPA, L-DOPA and SAA

L-DOPA and SAA levels were measured through high-performance liquid chromatography (HPLC) analysis, using a PerkinElmer system (Flexar Technology Limited, USA) with a SunFire column (4.6 × 250 mm, 5 μm) (Waters, Milford, MA, USA). After centrifugation, the supernatant of each mixture was collected and 10 μL injected into the HPLC system. The mobile phase contained a mixture of methanol and 0.1% formic acid solution, at a flow rate of 1 mL min^−1^, and a column temperature of 35 °C. A linear gradient, 10–100% methanol, was performed over 8 min, followed by a wash with 100% methanol for 10–15 min and recalibration with 10% methanol for 15–20 min. L-DOPA and SAA were detected using an ultraviolet detector at a wavelength of 280 nm.

The samples for DHPPA detection were centrifuged and filtered through a 0.22-μm filter membrane, separated using an Aminex HPX-87H column (300 × 7.8 mm, 9 μm) (Bio-Rad, Hercules, CA, USA), eluted with 5 mM H_2_SO_4_ (0.6 mL min^−1^, 35 °C), and detected at 210 nm.

The samples for ee value SAA detection were centrifuged, the supernatants filtered using a 0.22-μm filter membrane, and 4 μL were injected into an Agilent 1260 HPLC (Agilent, Santa Clara, CA, USA) with a CHIRALPAK IG-3 column (250 × 4.6 mm, 3 µm) (Daicel Co., Osaka, Japan), and an UV detector at 205 nm. The mobile phase comprised a trimethylamine (0.1%, pH 3.0):methanol 4:6 solution, at a flow rate of 0.2 mL min^−1^ and a column temperature of 25 °C.

## Results

### Design of a tri-enzymatic cascade pathway to synthesize SAA in vivo

As illustrated in Fig. 2a, SAA synthesis from L-DOPA comprises two steps: first, L-DOPA undergoes oxidative deamination by L-amino acid deaminase (LAAD, EC 1.4.3.2) to produce the prochiral intermediate 3,4-Dihydroxyphenylpyruvic acid (DHPPA); second, DHPPA undergoes reduction by NADH-dependent α-keto acid reductase to generate SAA, with coupling of formate dehydrogenase (FDH, EC 1.2.1.2) for NADH regeneration. For this process, three genes were amplified, overexpressed, and purified (Additional file 1: Fig. S1a–b). A previously reported highly active LAAD mutant from *Proteus mirabilis* (*Pm*LAAD^**M2**^, H295S/V437S) (Wu et al. 2021) and phenylpyruvate reductase from *Lactobacillus* sp. CGMCC 9967 (*La*PPR) (Xu et al. 2016) were selected based on the results of measuring their specific activity (Additional file 1: Table S3–S4); and the FDH from *Candida boidinii* (*Cb*FDH)was used to regenerate NADH. To assess the feasibility of the cascade pathway in vitro, these three enzymes were combined in an equimolar ratio and incubated with 1 g L^−1^ L-DOPA, 0.5 mM NAD^+^, and 1 g L^−1^ sodium formate for 1 h, after which 0.62 ± 0.07 g L^−1^ SAA was detected (Additional file 1: Fig. S2). The identity of the final product was confirmed using liquid chromatography–mass spectrometry (LC–MS) (Additional file 1: Fig. S3). This result demonstrates that the designed cascade composed of *Pm*LAAD^**M2**^, *La*PPR, and *Cb*FDH successfully converts L-DOPA to SAA. The effect of the ratio of *Cb*FDH:*La*PPR (from 0.1:1 to 3:1) on the SAA titer was further investigated using 15 g L^−1^ DHPPA and *La*PPR activity fixed in 5 U mL^−1^. As illustrated in Fig. 2b, when *Cb*FDH:*La*PPR ratio reached 0.3:1, the SAA titer increased to 13.9 ± 0.4 g L^−1^, with 91.7% conversion rate. Similarly, based on the enzyme activity ratio of *Cb*FDH:*La*PPR was maintained at 0.3:1, *Pm*LAAD^**M2**^, *Cb*FDH and *La*PPR were added to the reaction system together, when *Pm*LAAD^**M2**^: *La*PPR ratio was set to 0.6:1, the SAA titer was 12.9 ± 0.4 g L^−1^, with 85.6% conversion rate (Fig. 2c). Therefore, the optimal *Pm*LAAD^**M2**^:*La*PPR:*Cb*FDH ratio was defined to 0.6:1:0.3.

**Fig. 3:**
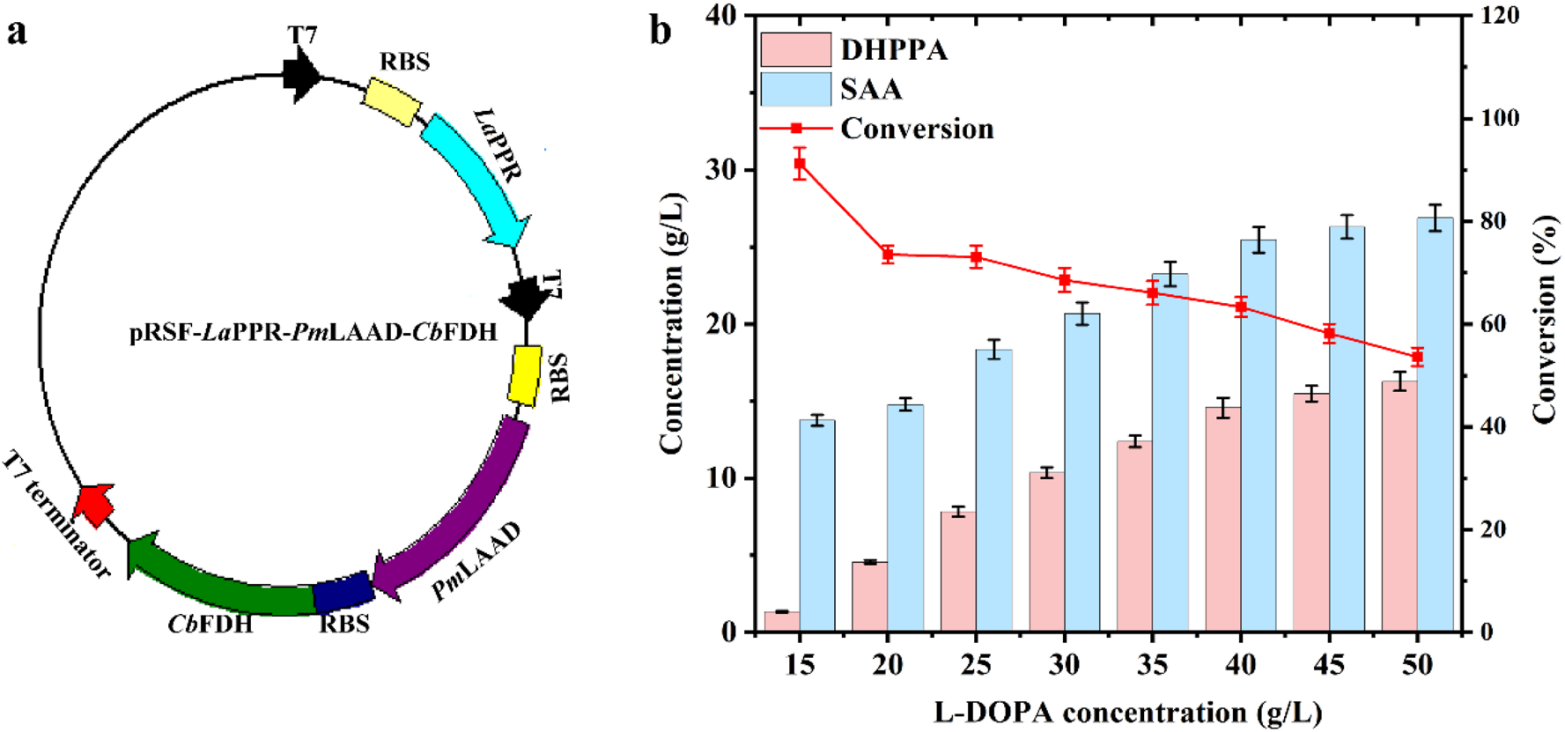
The construction and evaluation of *E. coli* YJH01. a Map of plasmid used to create strain *E. coli* YJH01. b The transformation performance of *E. coli* YJH01 with 20 g L^−1^ whole-cell catalysts, 15–50 g L^−1^ L-DOPA and 16–60 g L^−1^ sodium formate in Tris–HCl buffer (50 mM, pH 7.0, containing 5 mM NAD^+^) in 30 ℃ for 12 h. The data represent mean ± s.d., as determined from three independent experiments

To construct a highly efficient conversion system for industrial application, three enzymes were co-expressed in one host strain. Then, the genes *Pm*LAAD^**M2**^, *La*PPR, and *Cb*FDH were inserted into the plasmid pRSFDuet-1 (Fig. 3a) and transformed in *Escherichia coli* BL21 (DE3), resulting in strain *E. coli* YJH01. The expression of these three enzymes was verified using SDS-PAGE (Additional file 1: Fig. S1). The conversion performance of *E. coli* YJH01 was investigated with 20 g L^−1^ wet cells (Fig. 3b). When L-DOPA concentration was increased from 15 g L^−1^ to 50 g L^−1^ (meanwhile, the concentration of co-substrate sodium formate ranged from 16 g L^−1^ to 60 g L^−1^), the SAA titer increased from 12.8 ± 0.36 g L^−1^ to 26.9 ± 0.85 g L^−1^, while the conversion rate decreased from 91.2% to 53.6%, respectively. Concurrently, the intermediate DHPPA increased from 1.4 ± 0.05 g L^−1^ to 16.3 ± 0.61 g L^−1^, suggesting insufficient *La*PPR activity. The comparison between the in vivo activities of *Pm*LAAD^**M2**^ (145 ± 3.7 U mL^−1^), *La*PPR (36.2 ± 1.1 U mL^−1^) and *Cb*FDH (357 ± 10.2 U mL^−1^) revealed *La*PPR as the bottleneck in this pathway, at the ratio of 4.0:1:9.9 (Additional file 1: Table. S5). Therefore, improving the enzymatic activity of *La*PPR is crucial for enhancing SAA production.

### Crystal structure and catalytic mechanism of *La*PPR

**Fig. 4:**
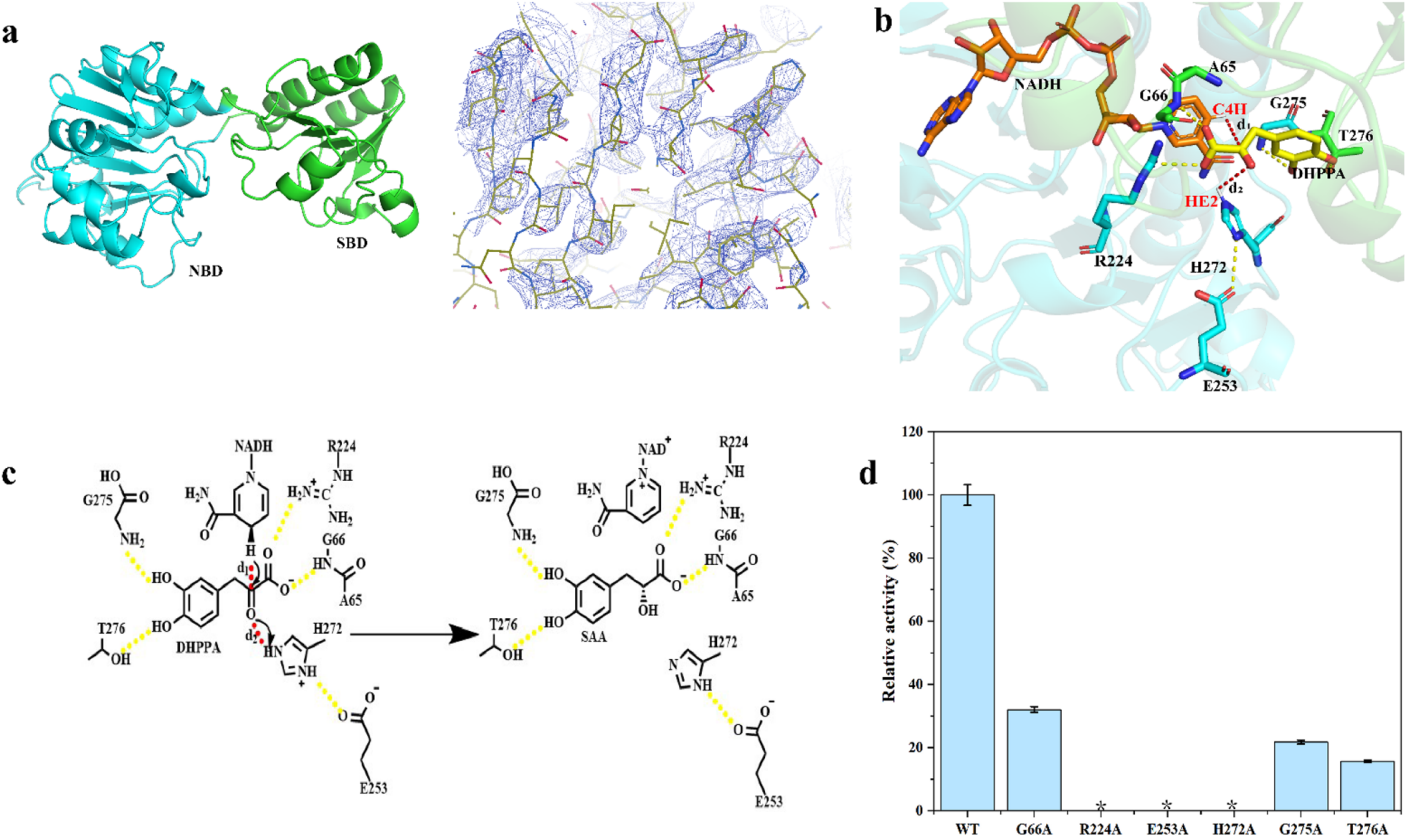
Crystal structure and catalytic mechanism of *La*PPR. **a** X-ray crystal structure and electron density map of apo-*La*PPR. **b** Docking binding model of NADH and DHPPA into the active site of *La*PPR. **c** The catalytic mechanism of *La*PPR. Hydrogen bonds are shown in gold dashed lines. d Alanine scanning of key residues. Asterisks indicate that the activity was not measurable. The data represent mean ± s.d., as determined from three independent experiments

The catalytic mechanism of *La*PPR was investigated to improve its enzymatic activity. First, we solved the crystal structure of apo-*La*PPR (PDB ID: 8HPG) (Fig. 4a**, **Additional file 1**: **Fig. S5). It exists as a homodimer, and each monomer folds into two distinct domains: the substrate-binding domain (SBD) and the nucleotide-binding domain (NBD). The SBD is mostly formed by 88 N-terminal residues, which folds into four α-helices and five β-strands, as well as the corresponding connecting loops. The NBD is formed by intervening residues (89–275), which fold into six central parallel β-strands surrounded by seven α-helices. The active site is located at the base of the interface between the two domains, and the two active sites of the dimer are ~ 35 Å apart. We conducted several attempts to obtain the X-ray crystal structure of *La*PPR in complex with NADH and DHPPA, through optimization of the protein concentration, pH value, temperature, and precipitant concentration, and even attempted rescreening for new crystal conditions; however, we were not successful. Alternatively, we constructed the *La*PPR–NADH binary complex by overlapping with the crystal structure of the complex (PDB ID: 2EKL) (Singh et al. 2014). Subsequently, DHPPA was docked into the active site of the *La*PPR–NADH binary complex to obtain a kinetically stable *La*PPR–NADH–DHPPA ternary complex by molecular dynamics (MD) simulations (Fig. 4b). The carboxyl group of DHPPA forms hydrogen bonds with the side chain guanidine group of R224 and with the main chain amide groups of A65 and G66. The two hydroxyl groups of DHPPA also form hydrogen bonds with G275 and T276. The conserved residues R224, E253 and H272 (Additional file 1: Fig. S6) may work as catalytic triad; H272 is suggested as a general acid to protonate the carbonyl carbon of DHPPA, and E253 could form a charge relay system with H272 for proton transfer. Based on the reported mechanism underlying the 2-hydroxy acid dehydrogenase subfamily (Jia et al. 2018; J Zhou et al. 2020), we proposed a catalytic mechanism for *La*PPR: a hydride is transferred from the nicotinamide moiety carbon C_4_H of NADH to the carbonyl carbon atom of DHPPA, while a proton is transferred from H272 to the carbonyl oxygen atom of DHPPA to produce SAA (Fig. 4c). To verify the functions of the above residues, we site-directed mutated G66, R224, E253, H272, G275 and T276 to alanine (Fig. 4d). R224A, E253A and H272A completely abolished activity, whereas G66A, G275A and T276 decreased the activity by > 50%.

**Fig. 5:**
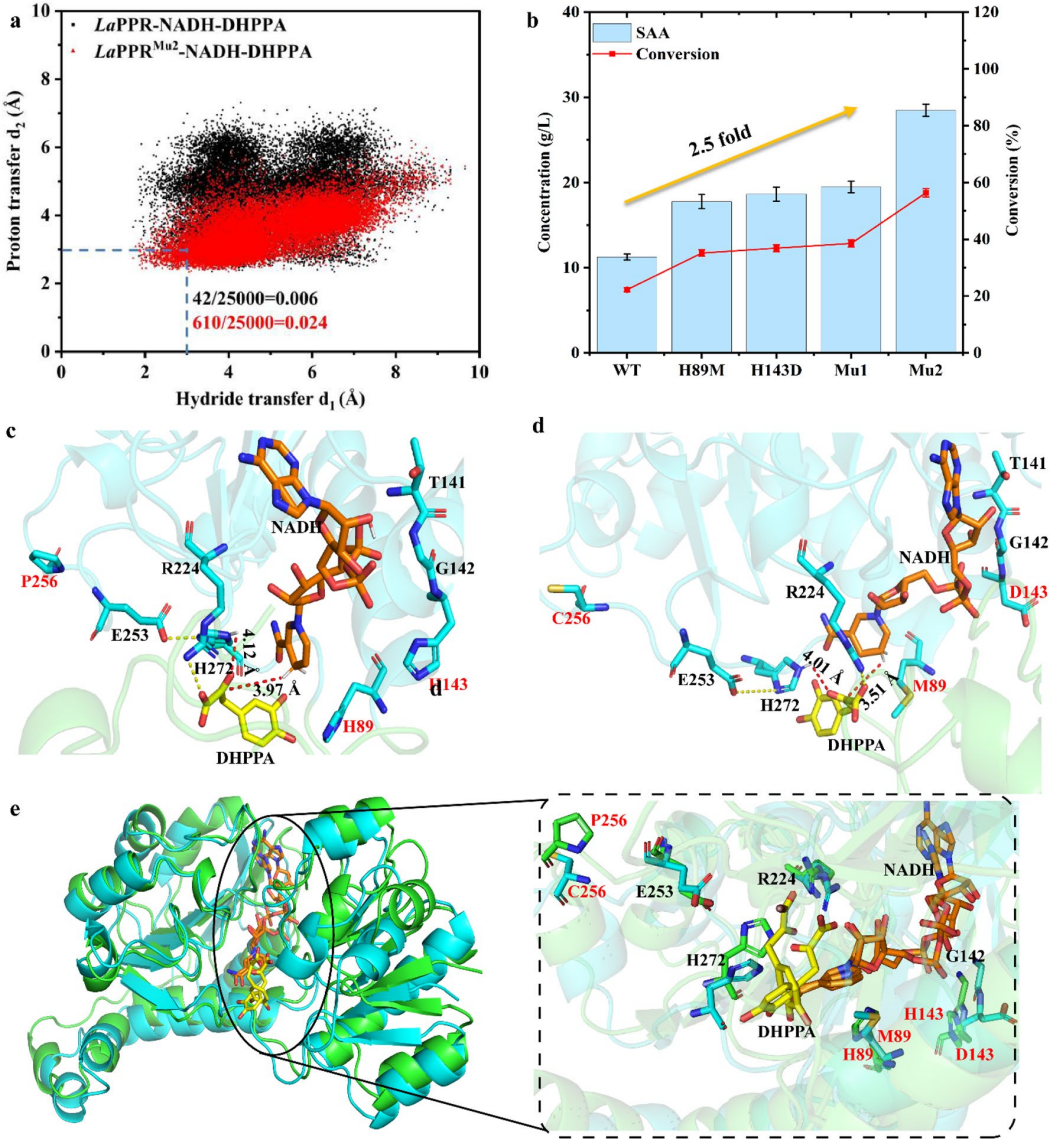
Protein engineering to enhance the activity of *La*PPR. **a** MD plots for the proportion of the catalytically active conformations (*d*_1_ ≤ 3.0 and *d*_2_ ≤ 3.0, dotted blue box) to the total conformation on the *La*PPR–NADH–DHPPA and *La*PPR^**Mu2**^–NADH–DHPPA complex during 50 ns MD simulations. **b** The conversions were performed in 10 mL Tris–HCl buffer (50 mM, pH 7.0, containing 3 mM NAD^+^, 50 g L^−1^ DHPPA and 60 g L^−1^ sodium formate) with 20 g L^−1^ *La*PPR and *Cb*FDH wet whole-cell biocatalysts in 30 °C for 12 h. Representative MD snapshots of **c** *La*PPR–NADH–DHPPA and **d** *La*PPR^**Mu2**^–NADH–DHPPA complexes. The initial structure of *La*PPR^Mu2^ was predicted from AlphaFold. **e** Structural alignment of *La*PPR–NADH–DHPPA (green) and *La*PPR^**Mu2**^ NADH–DHPPA (cyan) complexes

According to the mechanism of *La*PPR, two key distances were defined: the hydride transfer d_1_ describing the distance between the carbonyl carbon atom of DHPPA and the C4H hydrogen atom of NADH, and the proton transfer d_2_ as the distance between the carbonyl oxygen atom of DHPPA and the imidazole ring HE2 hydrogen atom of H272. However, MD simulations on *La*PPR–NADH–DHPPA ternary complex showed that, among 25,000 snapshots, only 42 frames were in catalytically active conformation, with both d_1_ and d_2_ smaller than 3.0 Å (Fig. 5a). Furthermore, d_1_ was of 3.97 ± 0.74 Å and d_2_ of 4.12 ± 0.46 Å (Additional file 1: Fig. S8), suggesting that longer d_1_ and d_2_ difficult hydride and proton transfer, ultimately resulting in lower enzyme activity. Therefore, shorter d_1_ and d_2_ values are expected to improve the activity of *La*PPR.

### Protein engineering to enhance the activity of *La*PPR

To shorten d_1_ and d_2_, 24 residues near the active site were selected as potential mutation sites. First, 16 residues near R224 and H272 were selected for NNK site saturation mutagenesis (SSM). Among those mutants, the SAA titer of the two single mutants H89M and H143D increases from 11.3 g/L (wild type) to 17.8 g/L and 18.6 g/L, respectively (Fig. 5b). When combining these two single mutants to obtain the double mutant *La*PPR^**Mu1**^ (H89M/H143D), the SAA titer increased to 19.5 g/L. (Fig. 5b) Furthermore, MD simulations showed that the flexible regions of loop-7 (residues 249–257) tended to approach the active site. Eight residues in the loop-7 were selected to further improve the activity of *La*PPR^**Mu1**^ (H89M/H143D). The mutant *La*PPR^**Mu2**^ (H89M/H143D/P256C) exhibited 2.5-fold SAA titer that of WT (Fig. 5b). The kinetic parameters of *La*PPR and its mutants are summarized in Table 1. The specific activity, *K*_m_, *k*_cat_/*K*_m_ of *La*PPR^**Mu2**^ were 3.8 times, 0.6-fold, 10.3 folds the corresponding values of **WT**.

**Table 1:** Kinetic parameters of the purified *La*PPR WT and its mutants

Mutants	Specific activity (U mg^−1^)	*K*_m_ (mM)	*k*_cat_ (s^−1^)	*k*_cat_/*K*_m_ (s^−1^ mM^−1^)
*La*PPR^WT^	5.8 ± 0.2 (1)	6.20 ± 0.15	1.21 ± 0.03	0.20 (1)
*La*PPR^H89M^	9.1 ± 0.3 (1.6)	5.74 ± 0.08	1.37 ± 0.06	0.24 (1.2)
*La*PPR^143D^	9.3 ± 0.3 (1.6)	5.03 ± 0.14	2.08 ± 0.11	0.41 (2.1)
*La*PPR^Mu1^	11.4 ± 0.3 (2.0)	3.63 ± 0.17	2.35 ± 0.12	0.65 (3.3)
*La*PPR^Mu2^	21.8 ± 0.7 (3.8)	2.52 ± 0.27	5.74 ± 0.24	2.05 (10.3)

Exhaustive efforts to obtain the crystal structure of *La*PPR^**Mu2**^ with high resolution by optimizing protein/ligand concentrations, pH values, temperatures, precipitant concentration, or even rescreening the crystal condition were not successful. Therefore, the initial structure of *La*PPR^**Mu2**^ was predicted by AlphaFold and aligned with the crystal structure of *La*PPR showed that the (root mean square deviation) RMSD value was 0.261 Å for 312 C_α_ atoms (Additional file 1: Fig. S7). To elucidate the molecular basis of higher activity of *La*PPR^**Mu2**^, we conducted MD simulations on the *La*PPR^**Mu2**^–NADH–DHPPA ternary complex. As expected, the proportion of catalytically active conformations in the mutant increased (610/25000 vs 42/25000, Fig. 5a). Furthermore, the hydride transfer d_1_ and proton transfer d_2_ shortened from 3.97 ± 0.74 Å to 3.51 ± 0.61 Å and from 4.12 ± 0.46 Å to 4.01 ± 0.56 Å, respectively (Additional file 1: Fig. S8), suggesting that hydride and proton transfer are more likely to occur, which is consistent with the measured increase in *k*_cat_. Upon the mutation of H143D, anticorrelated motions from the increased interactions between the phosphate group in NADH and the nearby residues G142 and T141 might have a pushing motion, which pushes the C4H of NADH niacinamide ring toward the Cα atom of DHPPA (Fig. 5c–e). When H89 was mutated to methionine, the adverse interaction of nearby residues around the substrate seems to reduced, allowing the C_α_ atom of DHPPA to be closer to NADH and H272. Additionally, the P256C mutation likely contributed to the formation of a larger catalytic pocket (Additional file 1: Fig. S9) and a more flexible conformation (Additional file 1: Fig. S8), which modified the binding model of DHPPA, thus making DHPPA closer to NADH and H272. These results suggest that the substrate-binding patterns and internal interactions in *La*PPR^**Mu2**^ may be altered and need further investigation.

### One-pot production of SAA in vivo

**Fig. 6:**
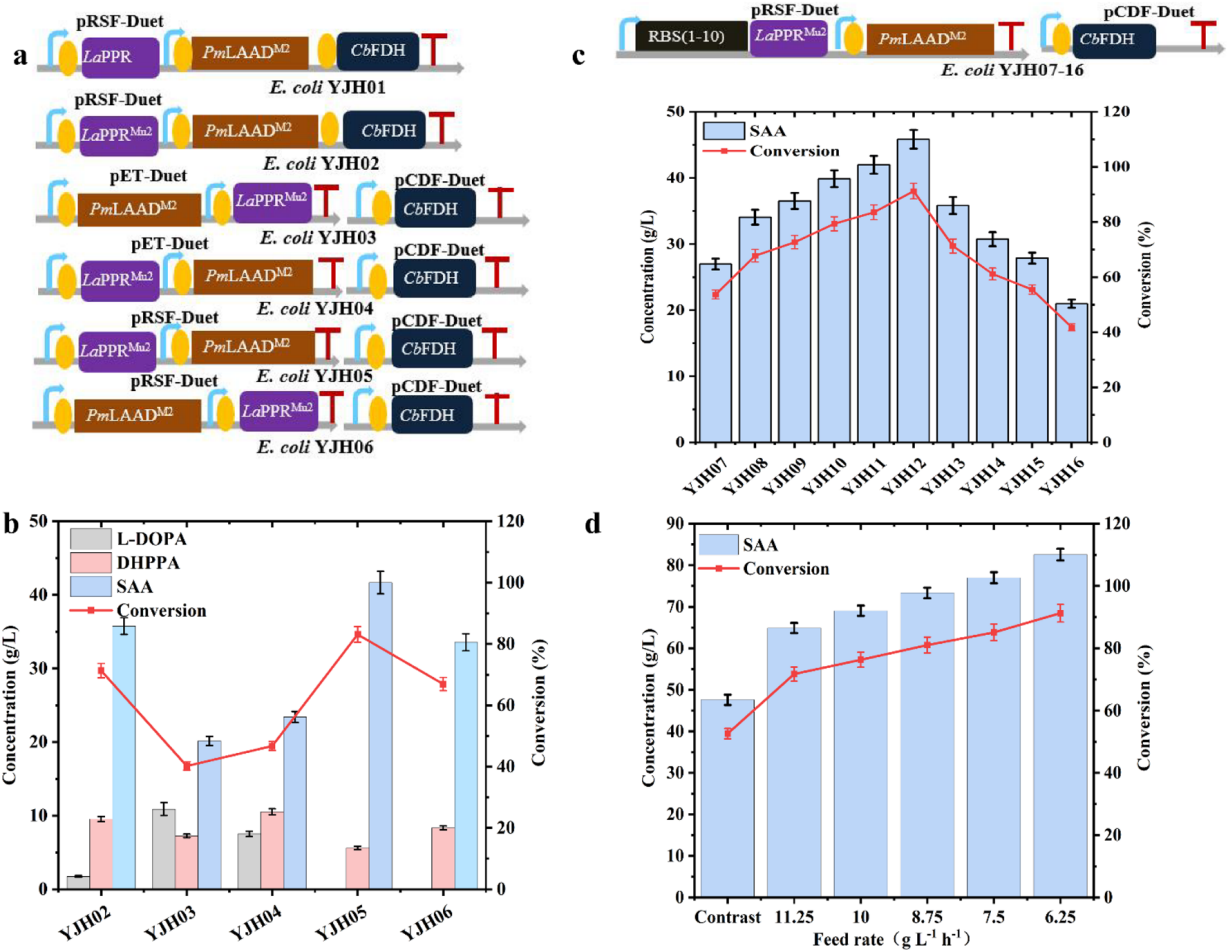
One-pot production of SAA in vivo. **a** The gene organization in recombinant strains. Effect of copy number plasmids (**b**) and RBS (**c**) on SAA production with 50 g L^−1^ L-DOPA and 20 g L^−1^ wet cell in 10 mL Tris–HCl buffer (50 mM, pH 7.0, containing 3 mM NAD^+^ and 60 g L^−1^ sodium formate) in 30 °C for 12 h. **d** Effect of feed rate of L-DOPA on SAA production with 90 g L^−1^ L-DOPA (total) and 30 g L^−1^ wet cell in 100 mL Tris–HCl buffer (50 mM, pH 7.0, containing 0.6 mM NAD^+^ and 108 g L^−1^ sodium formate) in 30 °C for 12 h. The data represent mean ± s.d., as determined from three independent experiments. The substrate non-batch feeding mode was used as a contrast

We imported the best triple mutant *La*PPR^**Mu2**^ into the cascade pathway in vivo to generate the resulting strain *E. coli* YJH02 (Fig. 6a), and it produced 35.8 g L^−1^ SAA from 50 g L^−1^ L-DOPA (Fig. 6b). However, 1.75 g L^−1^ L-DOPA remained unmodified and 9.54 g L^−1^ DHPPA accumulated. This could derive from the specific activity of *Pm*LAAD^**M2**^, *La*PPR^**Mu2**^ and *Cb*FDH in *E. coli* YJH02, being 134 ± 3.5 U mL^−1^, 63.2 ± 2.6 U mL^−1^, and 310 ± 8.7 U mL^−1^, respectively (Additional file 1: Table. S5). These values correspond to an enzymatic activity ratio of 2.1:1:4.9, suggesting that high *Cb*FDH activity affected the expression of *Pm*LAAD^**M2**^ and *La*PPR^**Mu2**^. To overcome this issue, the recombinant strains were constructed using different combinations with different copy number plasmids (pCDFDuet-1, pETDuet-1, and pRSFDuet-1 plasmids with copy numbers of 20, 40, and 100, respectively) (Fig. 6a). Among them, *E. coli* YJH05 was found to be best for SAA production (Fig. 6b). In this strain, the activities of* Cb*FDH and *Pm*LAAD^**M2**^ were decreased by 42.3% (179 ± 4.3 U mL^−1^) and 4.4% (128 ± 3.2 U mL^−1^), while the activity of *La*PPR^**Mu2**^ was increased by 38.6% (87.6 ± 2.8 U mL^−1^) in *E. coli* YJH05 compared to the *E. coli* YJH02 (Additional file 1: Table. S5)*.* Whole-cell catalysis in *E. coli* YJH05 produced 41.7 g L^−1^ SAA from 50 g L^−1^ L-DOPA, corresponding to 83.1% conversion rate, and 5.62 g L^−1^ DHPPA was detected (Fig. 6b). Subsequently, ten ribosome-binding site (RBS) sequences with different translation rates were predicted from RBS Calculator v2.1 (https://salislab.net/software/predict) to regulate the expression level of *La*PPR (Additional file 1: Table. S6). Among the RBS strains, *E. coli* YJH12 with RBS6 produced a SAA titer of 45.8 g L^−1^, with a conversion rate of 91.2%, minimal DHPPA accumulation (0.86 g L^−1^) and no detectable remaining L-DOPA (Fig. 6c). The specific activities of *Pm*LAAD^**M2**^ and *Cb*FDH in this strain were 94.4 U mL^−1^ and 139 U mL^−1^, respectively, whereas the activity of *La*PPR^**Mu2**^ was 112 U mL^−1^, resulting in a ratio of 0.8:1:1.2 (*Pm*LAAD^**M2**^: *La*PPR: *Cb*FDH) (Additional file 1: Table. S5), suggested that the balance between *Pm*LAAD^**M2**^, *La*PPR^**Mu2**^ and *Cb*FDH was unobstructed. To further improve the production efficiency of SAA, the transformation conditions for *E. coli* YJH12 (Additional file 1: Fig. S10). The effect of pH (6.5 to 9.0), temperature (20 to 40 °C), wet-cell concentration (10 to 40 g L^−1^) and NAD^+^ content (0.2 mM to 1.6 mM) on the SAA titer were investigated at a 10 mL scale. Under the optimal conditions (pH 7.0, 30 °C, 15 g L^−1^ wet cells, 0.6 mM NAD^+^), 47.1 g L^−1^ SAA can be produced from 50 g L^−1^ L-DOPA with a conversion rate of up to 93.8%.

Finally, we explored the scale-up transformation of SAA and the effect of substrate feeding modes on its titer. We used substrate non-batch feeding as contrast, among them, the batch feeding has the highest titer: the initial concentrations of L-DOPA was 15 g L^−1^ and the ratio of L-DOPA to sodium formate was set 1:1.2, then L-DOPA was added hourly to increase its concentration by 6.25 g L^−1^ for the duration of 12 batches (Fig. 6d). Finally, from 90 g L^−1^ L-DOPA (the optimal batch feeding) in 12 h under the optimal transformation conditions, we obtained 82.6 g L^−1^ SAA using 30 g L^−1^ *E. coli* YJH12 (wet cells), with a conversion rate of 91.3%, productivity of 6.88 g L^−1^ h^−1^ and excellent e.e value (99%). These results demonstrate the potential of the engineered strain *E. coli* YJH12, for the industrial production of SAA from L-DOPA.

## Discussion

The two major challenges in the synthesis of SAA are (1) the low activity of the rate-limiting enzyme *La*PPR and (2) an unbalanced enzyme levels within the cascade. In previous studies, the optimization of pathway enzymes, mainly focused on plasmid copy number, whereas in vivo enzyme activity was overlooked (Xiong et al. 2019a, b, c; Xiong et al. 2019a, b, c). Additionally, protein engineering of the rate-limiting enzyme for SAA synthesis from L-DOPA has been poorly studied in the past. Analysis of the catalytic mechanism of *La*PPR highlighted that longer catalytic distances might prevent the transfer of the hydride and proton, ultimately leading to lower enzyme activity. Through mechanism-guided protein engineering, shorten hydride transfer distance *d*_1_ and proton transfer distance *d*_2_ can increase the ratio of the catalytic conformation and improve enzyme activity. The optimal triple variant, *La*PPR^**Mu2**^ (H89M/H143D/P256C), promotes physical proximity between DHPPA, NADH, and H272 through the improvement of the flexibility of loop-7 (residues 249–257). This triple mutant shortens catalytic distances and increases the proportion of catalytically active conformations. Furthermore, the specific activity and *k*_cat_/*K*_m_ of *La*PPR^**Mu2**^ were 3.8- and 10.3-fold that of the wild type, respectively. A series of approaches were conducted to overcome the unbalanced levels of enzymes in the cascade, such as adjusting the gene copy number (F Wang et al. 2020), the promoter strength (Tan et al. 2021) and RBS binding efficiency(Qian et al. 2018). We regulated the expression of the three enzymes by optimizing different copy numbers plasmids with different combinations as well as RBS sequences, to ensure that the enzymatic activity ratio of the three enzymes in vivo was close to the optimal enzymatic activity ratio in vitro. Finally, we optimized the transformation conditions and substrate feeding modes to further improve the efficiency of SAA production. Compared with non-batch feeding, the concentration of SAA increased from 47.5 g L^−1^ to 82.6 g L^−1^ (the highest so far) by using the optimal substrate batch feeding under the same reaction conditions, with a 91.3% conversion rate, a productivity of 6.88 g L^−1^ h^−1^, and 99% ee value. Therefore, the batch-feeding strategy can maintain the stability of enzyme activity and improve the utilization rate of substrate, which has the potential for large-scale and efficient synthesis of target products.

Our method shows two main advantages when compared to existing methods, (1) a higher space–time yield and (2) lower production and separation costs. The highest SAA titer reported in the literature was 23.6 g L^−1^ in 12 h and space–time yield was only 1.96 g L^−1^ h^−1^ (Xiong et al. 2019a, b, c). In this study, we achieved an 82.6 g L^−1^ titer in 12 h and the productivity increased to 6.88 g L^−1^ h^−1^ (the highest value reported in the literature to thid date). Moreover, 30 g L^−1^ wet strain *E. coli* YJH12 was used, whereas Xiong et al. required 50 g L^−1^ strain and there was accumulation of a coproduct, 42.9 g L^−1^ gluconic acid, which resulted in higher production and separation costs (Xiong et al. 2019a, b, c).

In conclusion, through mechanism-guided protein engineering, pathway enzyme expression equilibration, transformation conditions modifications and substrate feeding protocol optimization, the highest titer of SAA in vivo was 82.6 g L^−1^ from 90 g L^−1^ L-DOPA (batch feeding) with a molar conversion rate of 91.3%, 99% ee value and a productivity of 6.88 g L^−1^ h^−1^. These findings represent a potentially attractive strategy for the industrial production of SAA.

### Supplementary Information

**Additional file 1**:** Table S1.** The primers used in this study.** Table S2.** Data collection and refinement statistics of apo-LaPPR crystal structure.** Table S3.** Specific enzyme activities of L-AAD from different organisms. ** Table S4.** Specific enzyme activities of α-keto acid reductase from different organisms. **Table S5.** Specific enzyme activities of PmLAAD, LaPPR and CbFDH in E. coli. **Table S6.** Types and sequences of different RBS. **Figure S1.** SDS-PAGE analysis. **a** The crude PmLAAD enzyme, purified LaPPR and purified CbFDH enzyme; **b** The purified PmLAAD enzyme; **c** Three enzymes PmLAAD, LaPPR and CbFDH co-expressed in E. coli YJH01. **Figure S2.** Detection of SAA product in vitro biosynthesis pathway. **a** Analysis of the standard sample and the retention time of SAA was 18.17 min; **b** Analysis of the reaction mixture in vitro, and SAA product was detected at 18.17 min. **Figure S3**. Characterization of pure SAA by LC-MS.m/z: C9H10O5 [M-H]-, 197. **Figure S4.** Detection of DHPPA intermediate in the reaction mixture by HPLC. **a** Standard sample of DHPPA, the retention time was 10.66 min; **b** Analysis of the remaining DHPPA intermediate in reaction mixture, and DHPPA was detected at 10.67 min. **Figure S5.** The asymmetric unit of LaPPR contains two essentially identical units. **Figure S6.** Multiple sequence alignment of LaPPR with homologous PGDH sequences from superfamily. **Figure S7.** **a** Structural alignment of LaPPRand LaPPRMu2; **b** Ramachandran plot of LaPPRMu2 structure. The initial structure of LaPPRMu2 was predicted from AlphaFold. **Figure S8.** **a** Root-mean-square-deviationcalculated from MD simulationsof LaPPR-NADH-DHPPA and LaPPRMu2-NADH-DHPPA complex. **b** B-factor calculated from MD simulations of LaPPR-NADH-DHPPA and LaPPRMu2-NADH-DHPPA complex. **c** Distances of hydride transfer and proton transfercalculated from 50 ns MD simulations on the LaPPR-NADH-DHPPA complex. Distances of hydride transfer and proton transfercalculated from 50 ns MD simulations on the LaPPRMu2-NADH-DHPPA complex. Mean ± standard deviation is shown for 25000 snapshots of the 50 ns MD simulations. The initial structure of LaPPRMu2 was predicted from AlphaFold.** Figure S9.** **a** The wild-type LaPPR pocket, shown in yellow, has a volume size of 989 Å3; **b** The mutant LaPPRMu2 pocket, shown in yellow, has a volume size of 1359 Å3. The pocket volume for each of the structures has been computed with the POCASA server. The initial structure of LaPPRMu2 was predicted from AlphaFold. **Figure S10.** **a** Effect of conversion temperature on SAA concentration. The conversion reactions were performed in a 10 mL volume of 50 mM Tris-HCl buffer, pH 7.0, containing 3 mM NAD+, with 20 g L-1 wet whole-cell biocatalysts, 50 g L-1 L-DOPA and 60 g L-1 sodium formate, in 20–40 °C for 12 h. **b** Effect of conversion pH on SAA concentration. The conversion reactions were performed in a 10 mL volume of 50 mM Tris-HCl buffer, pH 6.5-9.0 containing 3 mM NAD+, with 20 g L-1 wet whole-cell biocatalysts, 50 g L-1 L-DOPA and 60 g L-1 sodium formate, in 30 °C for 12 h. **c** Effect of concentration of NAD+ on SAA concentration. The conversion reactions were performed in a 10 mL volume of 50 mM Tris-HCl buffer, pH 7.0, containing 0.2-1.6 mM NAD+, with 20 g L-1 wet whole-cell biocatalysts, 50 g L-1 L-DOPA and 60 g L-1 sodium formate, in 30 °C for 12 h.Effect of concentration of whole-cell catalyst on SAA concentration. The conversion reactions were performed in a 10 mL volume of 50 mM Tris-HCl buffer, pH 7.0, containing 0.6 mM NAD+, with 10-40 g L-1 wet whole-cell biocatalysts, 50 g L-1 L-DOPA and 60 g L-1 sodium formate, in 30 °C for 12 h. The data represent mean ± SD, as determined from three independent experiments.

## Data Availability

All data generated or analyzed during this study are included in this article.

## Abbreviations

SAA: Salvianic acid A
L-DOPA: L-Dihydroxyphenylalanine
DHPPA: Dihydroxy phenylpyruvic acid
L-AAD: L-Amino acid deaminase
PPR: Phenylpyruvate reductase
FDH: Formate dehydrogenase
MD: Molecular dynamics
SBD: Substrate-binding domain
NBD: Nucleotide-binding domain
