# Supplementary material for: Efficient production of salvianic acid A from l-dihydroxyphenylalanine through a tri-enzyme cascade
Source: Bioresour Bioprocess. 2023 May 1;10(1):31. doi: 10.1186/s40643-023-00649-0 (PMC10992476; doi:10.1186/s40643-023-00649-0)
Supplement: Supplementary file 1 — Additional file 1: Table S1. The primers used in this study. Table S2. Data collection and refinement statistics of apo-LaPPR crystal structure. Table S3. Specific enzyme activities of L-AAD from different organisms. Table S4. Specific enzyme activities of α-keto acid reductase from different organisms. Table S5. Specific enzyme activities of PmLAAD, LaPPR and CbFDH in E. coli. Table S6. Types and sequences of different RBS. Figure S1. SDS-PAGE analysis. a The crude PmLAAD enzyme, purified LaPPR and purified CbFDH enzyme; b The purified PmLAAD enzyme; c Three enzymes PmLAAD, LaPPR and CbFDH co-expressed in E. coli YJH01. Figure S2. Detection of SAA product in vitro biosynthesis pathway. a Analysis of the standard sample and the retention time of SAA was 18.17 min; b Analysis of the reaction mixture in vitro, and SAA product was detected at 18.17 min. Figure S3. Characterization of pure SAA by LC-MS.m/z: C9H10O5 [M-H]-, 197. Figure S4. Detection of DHPPA intermediate in the reaction mixture by HPLC. a Standard sample of DHPPA, the retention time was 10.66 min; b Analysis of the remaining DHPPA intermediate in reaction mixture, and DHPPA was detected at 10.67 min. Figure S5. The asymmetric unit of LaPPR contains two essentially identical units. Figure S6. Multiple sequence alignment of LaPPR with homologous PGDH sequences from superfamily. Figure S7. a Structural alignment of LaPPRand LaPPRMu2; b Ramachandran plot of LaPPRMu2 structure. The initial structure of LaPPRMu2 was predicted from AlphaFold. Figure S8. a Root-mean-square-deviationcalculated from MD simulationsof LaPPR-NADH-DHPPA and LaPPRMu2-NADH-DHPPA complex. b B-factor calculated from MD simulations of LaPPR-NADH-DHPPA and LaPPRMu2-NADH-DHPPA complex. c Distances of hydride transfer and proton transfercalculated from 50 ns MD simulations on the LaPPR-NADH-DHPPA complex. Distances of hydride transfer and proton transfercalculated from 50 ns MD simulations on the LaPPRMu2-NADH-DHPPA complex. Mean ± standard d [file 40643_2023_649_MOESM1_ESM.docx]

**Additional file Information**

**Efficient production of salvianic acid A from L-dihydroxyphenylalanine through a tri-enzyme cascade**

Jiahui Yang^1^, Wanqing Wei^2^, Changzheng Gao^3^, Wei Song^1^, Cong Gao^2^, Xiulai Chen^2^, Jia Liu^2^, Liang Guo^2^, Liming Liu^2^, Jing Wu^1*^

^1^ School of Life Science and Health Engineering, Jiangnan University, Wuxi 214122, China

^2^ State Key Laboratory of Food Science and Technology, Jiangnan University, Wuxi 214122, China

^3^ Department of Cardiology, Affiliated Hospital of Jiangnan University, Wuxi, 214122, China

*Corresponding author: Jing Wu

Mailing address: School of Life Science and Health Engineering, Jiangnan University, Wuxi 214122, China

Fax/Tel.: +86-510-85910059

E-mail: wujing@jiannan.edu.cn

This doc file includes:

Additional file 1: Table S1 to Table S6

Additional file 1: Figure S1 to Figure S10

# Additional file Tables

Additional file 1: **Table S1**. The primers used in this study

| **Name** | **Primer (5’-3’)** |
| --- | --- |
| Primers for gene amplification | |
| *La*PPR-F | ATCACCACAGCCAGGATCCGATGATGAAAATTCTGAAC |
| *La*PPR-R | CATTATGCGGCCGCAAGCTTTTAATAGCCGCGGGTCAGAT |
| *Pm*LAAD-F | AAGAAGGAGATATACATATGATGAACATTTCAAGGAGAA |
| *Pm*LAAD-R | GTTTCTTTACCAGACTCGAGTTACTTCTTAAAACGATCCA |
| *Cb*FDH*-*F | ATCACCACAGCCAGGATCCGATGAAGATCGTGTTAGTCCT |
| *Cb*FDH*-*R | CATTATGCGGCCGCAAGCTTTTATTTTTTATCGTGTTTCC |
| Primers for mechanism test | |
| G66A-F | GTGCAAGCGATGAGCGCGGCGGTGGATTATCTG |
| G66A-R | CAGCGGCAGATAATCCACCGCCGCGCTCATCGC |
| R224A-F | CTGTTTATTAACATTGGCGCGGGCCCGAGCGTG |
| R224A-R | CATATCCACGCTCGGGCCCGCGCCAATGTTAAT |
| E253A-F | CTGGATGTGGTTGATCCGGCGCCGCTGCCGCAA |
| E253A-R | GCTATCTTGCGGCAGCGGCGCCGGATCAACCAC |
| H272A-F | AACGTGCTGCTGACCCCGGCGATTAGCGGCACC |
| H272A-R | CGGCACGGTGCCGCTAATCGCCGGGGTCAGCAG |
| G275A-F | CTGACCCCGCATATTAGCGCGACCGTGCCGCAG |
| G275A-R | GCGCAGCTGCGGCACGGTCGCGCTAATATGCGG |
| T276A-F | ACCCCGCATATTAGCGGCGCGGTGCCGCAGCTG |
| T276A-R | GCGCAGCTGCGGCACCGCGCCGCTAATATGCGG |
| Primers for the site mutation | |
| H89-F | GCGAACACGAGCGGCATTNNKGCGGAACCG |
| H89-R | TTCCGCAATCGGTTCCGCMNNAATGCCGCT |
| H143-F | GTGATTTTTGGCACCGGCNNKATTGGCAGC |
| H143-R | CGCAATGGTGCTGCCAATMNNGCCGGTGCC |
| P256-F | GTTGATCCGGAACCGCTGNNKCAAGATAGC |
| P256-R | CCACAGCGGGCTATCTTGMNNCAGCGGTTC |
| H143D-F | GTGATTTTTGGCACCGGCGATATTGGCAGC |
| H143D-R | CGCAATGGTGCTGCCAATATCGCCGGTGCC |

Additional file 1: **Table S2.** Data collection and refinement statistics of apo-*La*PPR crystal structure.

|  | **apo-*LaPPR*** |
| --- | --- |
| **PDB code** | 8HPG |
| **Data collection** |  |
| Space group | P 1 21 1 |
| Cell dimensions |  |
| a, b, c (Å) | 89.89, 48.97, 89.89 |
| α, β, γ (°) | 90.00, 116.72, 90.00 |
| Resolution (Å) | 23.49-3.79 (3.79-3.89) |
| CC(1/2) | 0.748 |
| R_merge_ | 0.141(0.371) |
| R_pim_ | 0.062 |
| *I/σI* | 7.29(2.02) |
| Completeness (%) | 42.7(87.2) |
| **Refinement** |  |
| No. reflections | 6138 |
| R_work_/R_free_ | 0.25755/0.27950 |
| No. atoms | 9448 |
| B factors (Å^2^) | 47.2 |
| r. m. s. deviations |  |
| bond lengths (Å) | 0.0061 |
| bond angles (Å) | 1.5321 |

Additional file 1: **Table S3.** Specific enzyme activities of L-AAD from different organisms

| Enzymes | Organisms | Specific activity^a^  (U·mg^-1^ protein) |
| --- | --- | --- |
| *Pv*L-AAD | *Proteus vulgaris* | 7.1 ± 0.4 |
| *Pm*L-AAD^S98A^ | *Proteus mirabilis* | 8.3 ± 0.3 |
| *Pm*L-AAD^H295S/V437S^ | *Proteus mirabilis* | 9.4 ± 0.4 |
| *Pm*L-AAD^S98A/T105A/S106A/L341A^ | *Proteus mirabilis* | 7.7 ± 0.3 |
| *Pm*L-AAD^T105A/S412A/E417A/E340A/E145A^ | *Proteus mirabilis* | 8.2 ± 0.2 |

Note. L-AAD: L-amino acid deaminase

^a^ The specific activity was determined with 10 μM purified L-AAD and 10 mM L-DOPA in 1 mL Tris-HCl buffer (50 mM, pH7.0) at 30 °C for 10 min. The data represent mean ± SD, as determined from three independent experiments.

Additional file 1: **Table S4.** Specific enzyme activities of α-keto acid reductase from different organisms

| Enzymes | Organisms | Specific activity^a^  (U·mg^-1^ protein) |
| --- | --- | --- |
| *La*PPR | *Lactobacillus. sp. CGMCC9967* | 5.8 ± 0.3 |
| *Lf*DLDH | *Lactobacillus fermentum JN248* | 3.1 ± 0.1 |
| *So*HPPR | *Soleostemon scutellarioides* | 0.9 ± 0.1 |
| *Pl*HPPR | *Plectranthus scutellarioides* | 0.7 ± 0.1 |
| *Wi*PPR | *Wickerhamia fluorescens* | 0.6 ± 0.1 |
| *Sc*HPPR | *Scutellaria baicalensis* | n/a |

Note. DLDH: D-lactate dehydrogenase; HPPR: hydroxyphenylpyruvate reductase; PPR: phenylpyruvate reductase; n/a, not available.

^a^The specific activity was determined with 10 μM purified α-Keto acid reductase 10 mM DHPPA, and 0.5 mM NADH in 1 mL Tris-HCl buffer (50 mM, pH 7.0) at 30 ℃ for 5 min. The data represent mean ± SD, as determined from three independent experiments.

Additional file 1: **Table S5.** Specific enzyme activities of *Pm*LAAD, *La*PPR and *Cb*FDH in *E. coli*

| Strains | *Pm*LAAD activity(U·mL^-1^) | *La*PPR  activity(U·mL^-1^) | *Cb*FDH  activity(U·mL^-1^) |
| --- | --- | --- | --- |
| *E. coli* YJH01 | 145 ± 3.7 | 36.2 ± 1.1 | 357 ± 10.2 |
| *E. coli* YJH02 | 134 ± 3.5 | 63.2 ± 2.6 | 310 ± 8.7 |
| *E. coli* YJH05 | 128 ± 3.2 | 87.6 ± 2.8 | 179 ± 4.3 |
| *E. coli* YJH12 | 94.4 ± 2.9 | 112 ± 3.1 | 139 ± 4.2 |

*Pm*LAAD: Activity was determined with 20 g L^-1^ Whole-cell catalyst, 50 g L^-1^ L-DOPA, in 1 mL Tris-HCl buffer (50 mM, pH 7.0) at 30 ℃ for 5 min.

*La*PPR: Activity was determined with 20 g L^-1^ Whole-cell catalyst, 50 g L^-1^ DHPPA, 5mM NADH in 1 mL Tris-HCl buffer (50 mM, pH 7.0) at 30 ℃ for 5 min.

*Cb*FDH: Activity was determined with 20 g L^-1^ Whole-cell catalyst, 50 g L^-1^ HCOONa, 5mM NAD^+^ in 1 mL Tris-HCl buffer (50 mM, pH 7.0) at 30 ℃ for 5 min.

The data represent mean ± SD, as determined from three independent experiments.

Additional file 1: **Table S6**. Types and sequences of different RBS

| Types | Sequence | Translation rate | Recombinant  strains |
| --- | --- | --- | --- |
| RBS-1 | TACTAATATTTAACCAGCTTGGTAC | 105.38 | *E. coli* YJH07 |
| RBS-2 | GGCTAGATACTTGGGTTCTGTAA | 211.42 | *E. coli* YJH08 |
| RBS-3 | TTAATAAGGAGATATA | 301.05 | *E. coli* YJH09 |
| RBS-4 | CGCGTTCCAAAAAACTATAAACAAGCAC | 372.48 | *E. coli* YJH10 |
| RBS-5 | TCCCGTAATACAAAGTTTAGTC | 413.58 | *E. coli* YJH11 |
| RBS-6 | TAACCATTTACGGCCAGCTC | 466.23 | *E. coli* YJH12 |
| RBS-7 | TCAAAGTCAAAT ACAAAGTTAAAGTT | 536.71 | *E. coli* YJH13 |
| RBS-8 | TTTGTCAAATATAGCGGCCA ATAATTGT | 593.61 | *E. coli* YJH14 |
| RBS-9 | CGGTCCTACGGCCCCAGT | 734.14 | *E. coli* YJH15 |
| RBS-10 | TCTTAGTCTGCTACTGTTAATCAT | 826.77 | *E. coli* YJH16 |

Translation rates were predicted from RBS Calculator v2.1 (https://salislab.net/software/predict).

# Additional file Figures


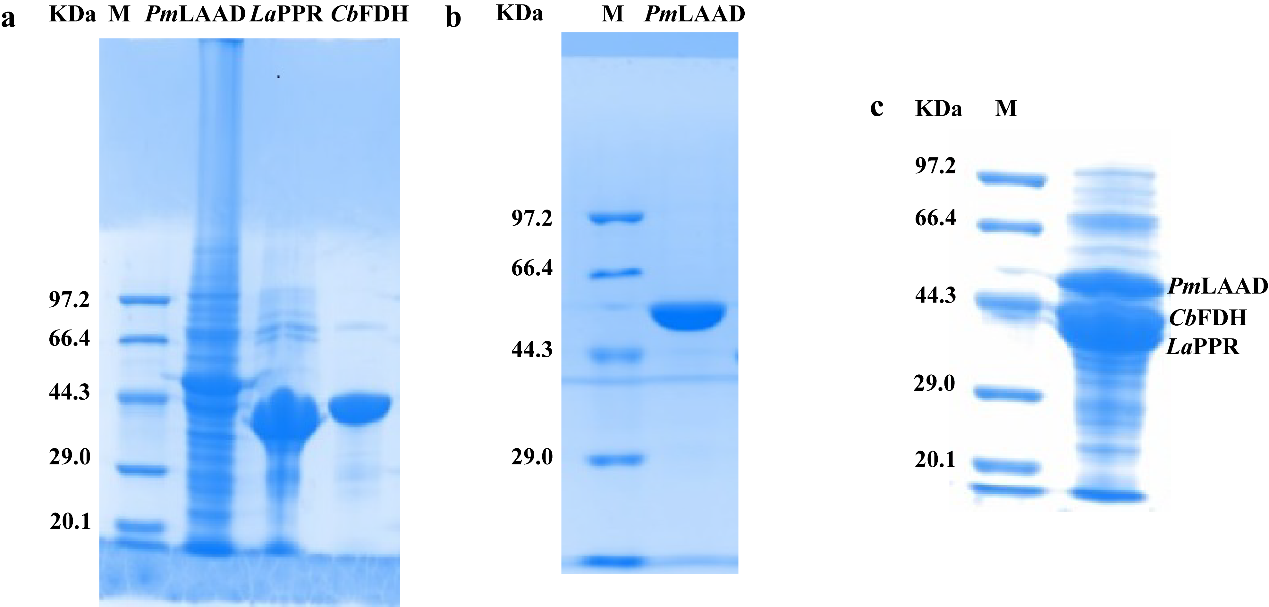


Additional file 1: Figure S1. SDS-PAGE analysis. (a) The crude *Pm*LAAD enzyme, purified *La*PPR and purified *Cb*FDH enzyme; (b) The purified *Pm*LAAD enzyme; (c) Three enzymes *Pm*LAAD, *La*PPR and *Cb*FDH co-expressed in E. coli YJH01.


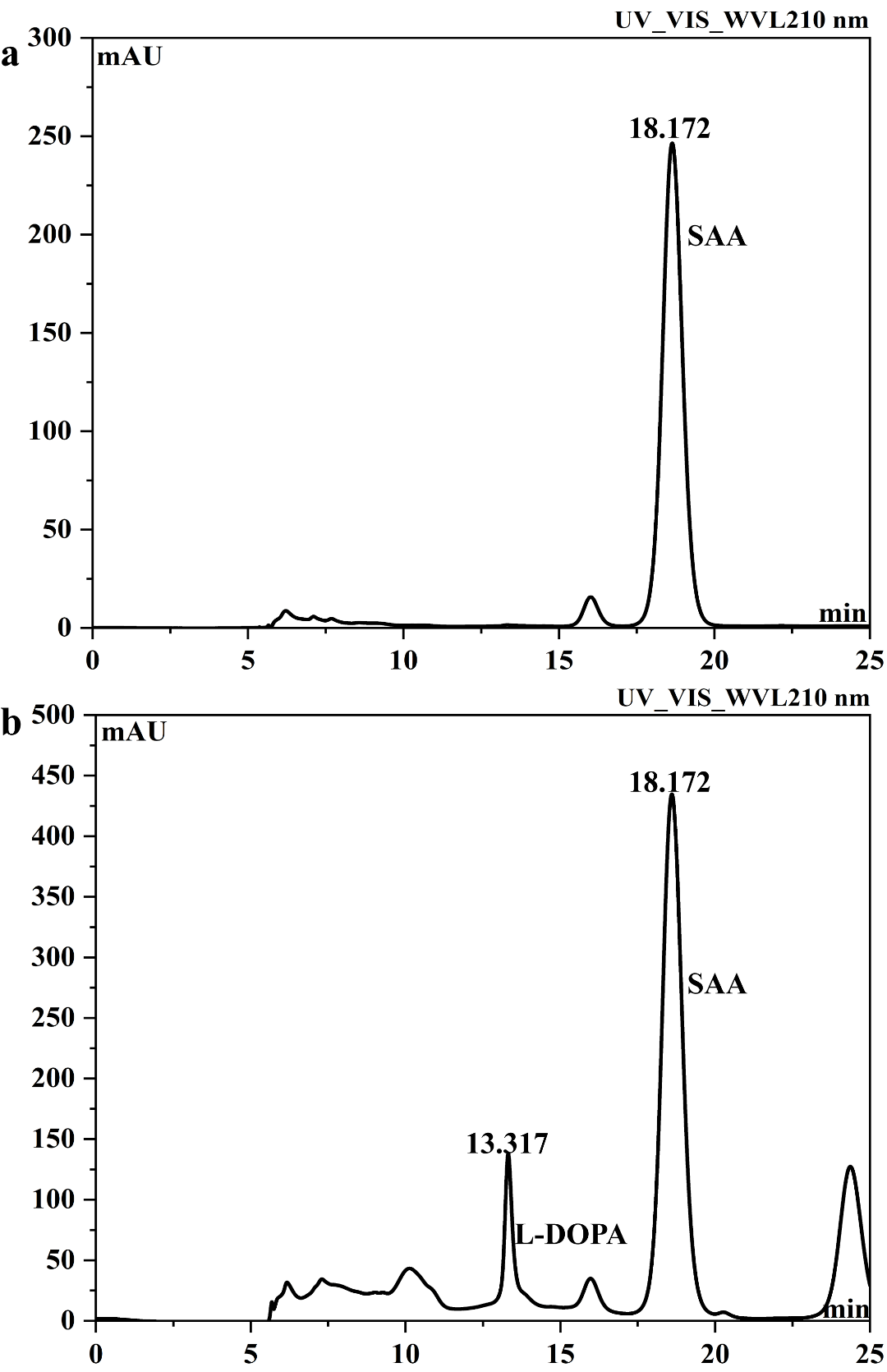


Additional file 1: **Figure S2.** Detection of SAA product *in vitro* biosynthesis pathway. (a) Analysis of the standard sample and the retention time of SAA was 18.17 min; (b) Analysis of the reaction mixture *in vitro*, and SAA product was detected at 18.17 min.


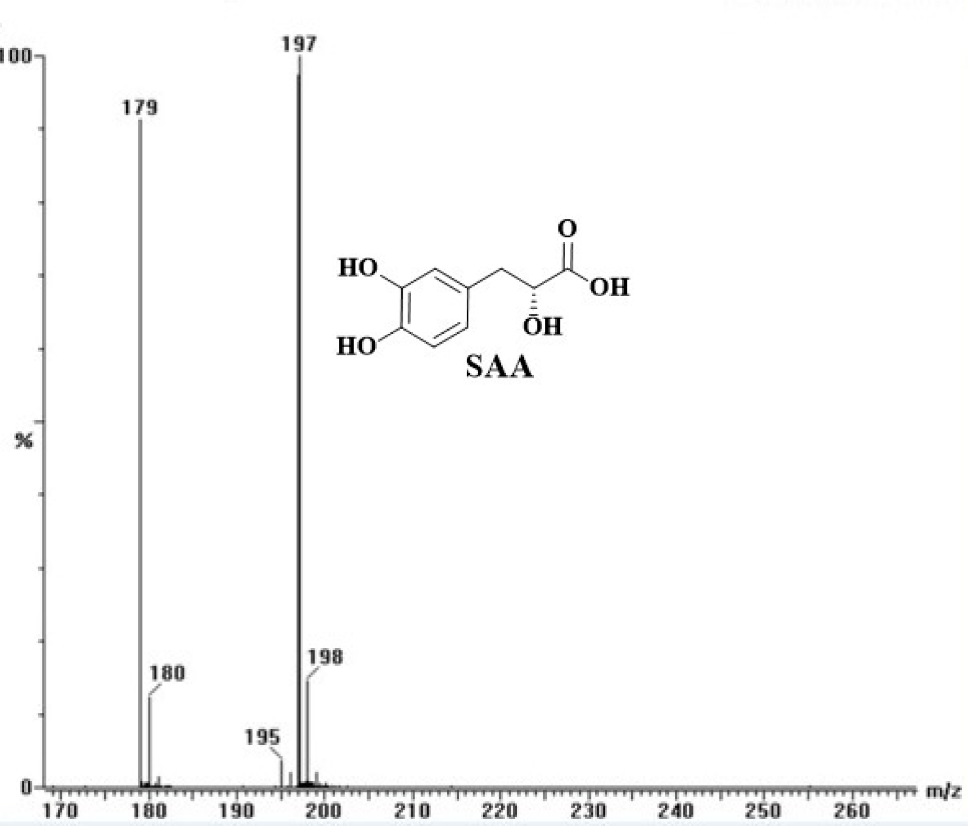


Additional file 1: **Figure S3.** Characterization of pure SAA by LC-MS. (ESI^-^) m/z: C_9_H_10_O_5_ [M-H]^-^, 197.


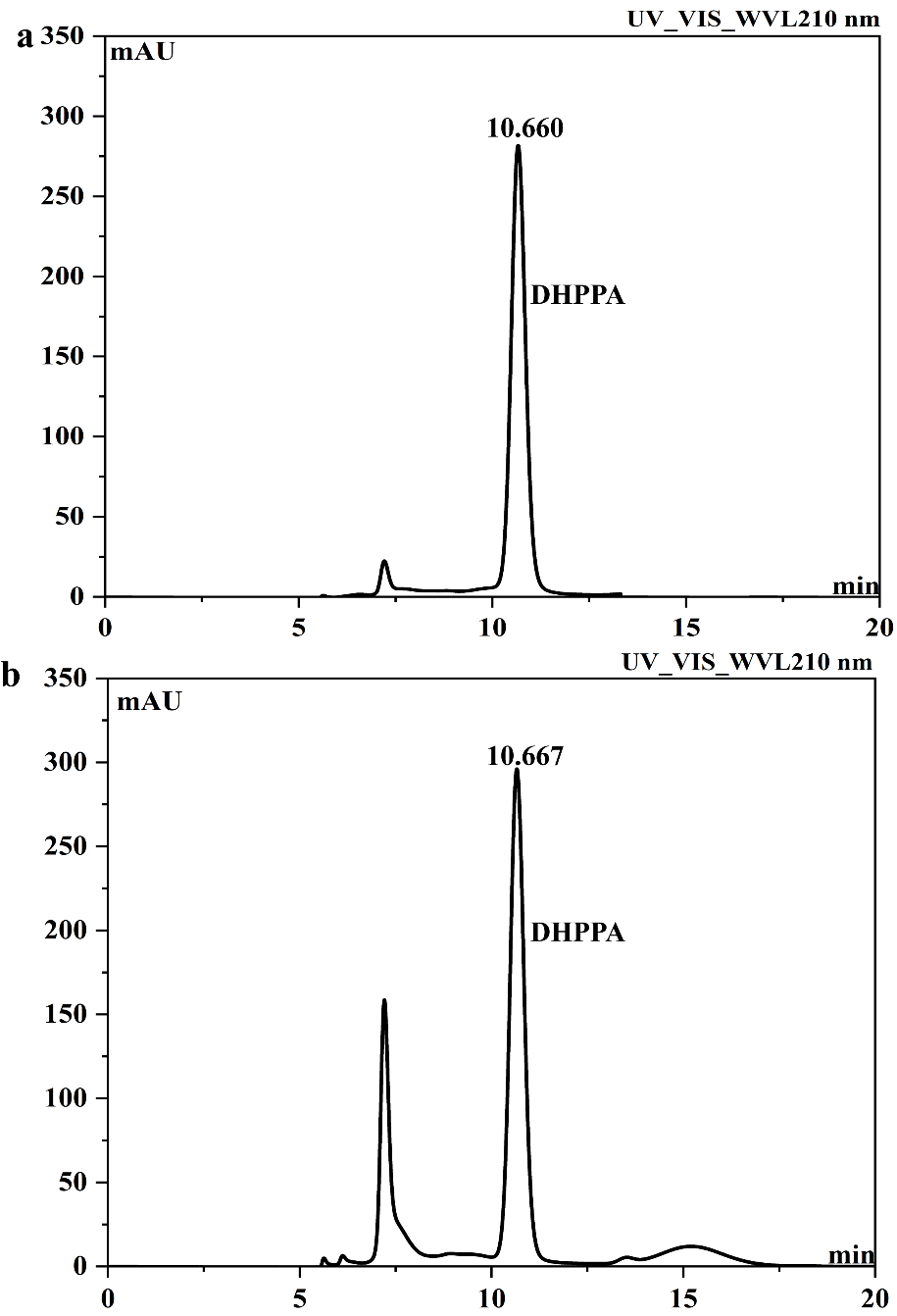


Additional file 1: **Figure S4.** Detection of DHPPA intermediate in the reaction mixture by HPLC. (a) Standard sample of DHPPA, the retention time was 10.66 min; (b) Analysis of the remaining DHPPA intermediate in reaction mixture, and DHPPA was detected at 10.67 min.

**
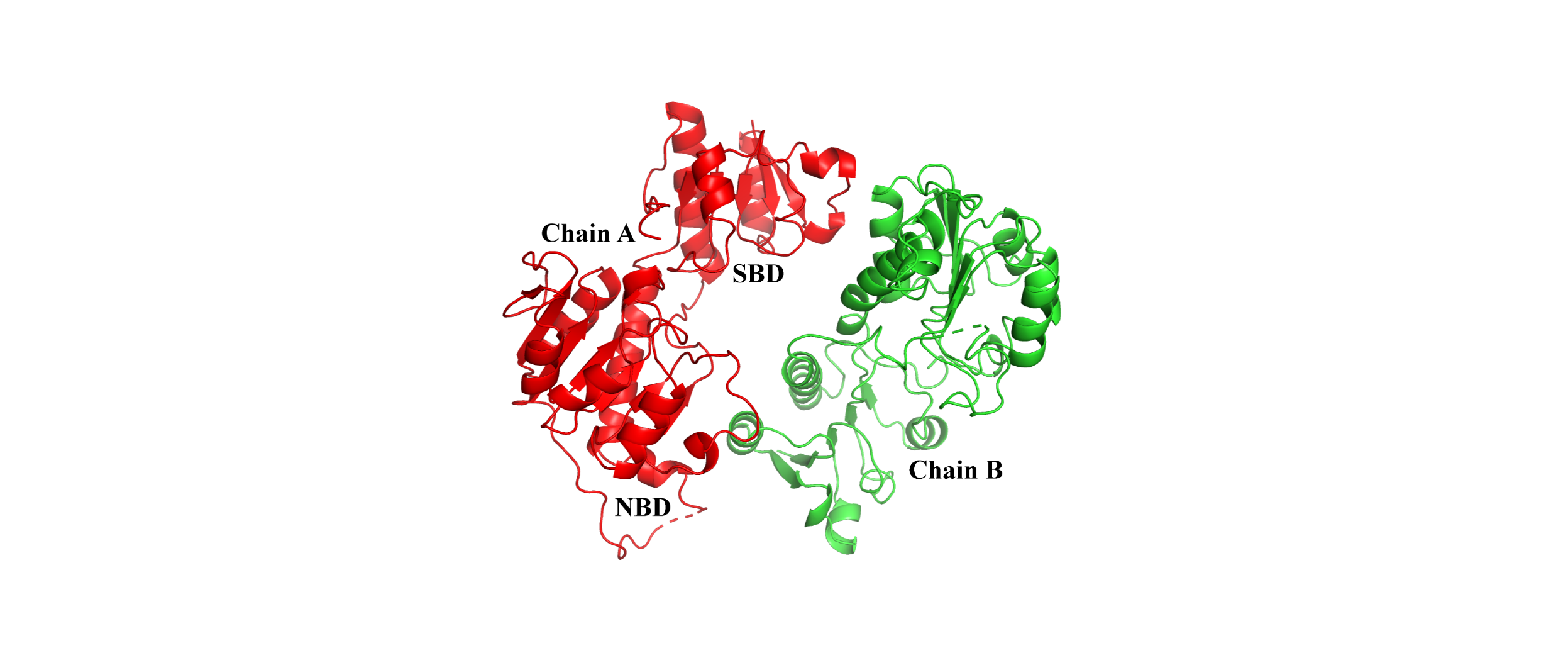
**

Additional file 1: **Figure S5.** The asymmetric unit of *La*PPR contains two essentially identical units.


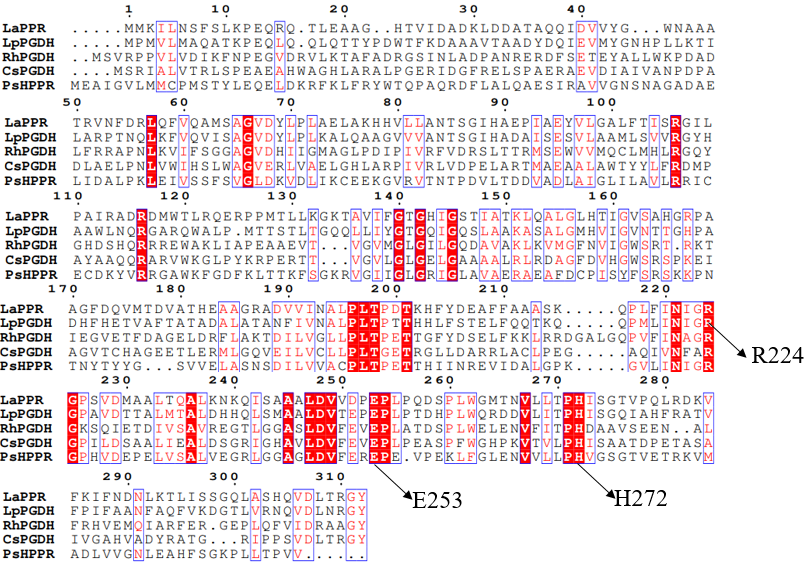


Additional file 1: **Figure S6.** Multiple sequence alignment of *La*PPR with homologous PGDH sequences from superfamily.


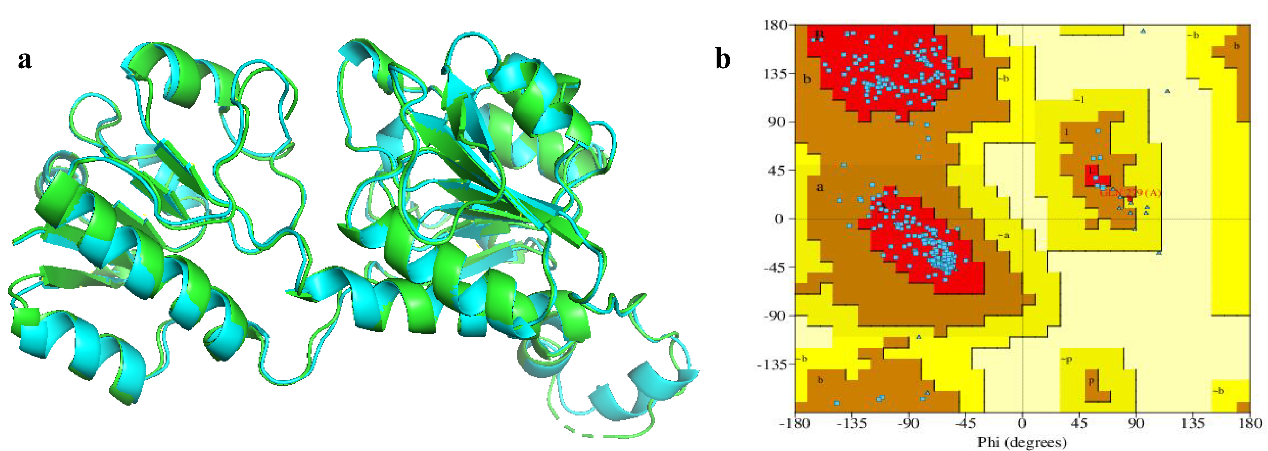


Additional file 1: **Figure S7.** (a) Structural alignment of *La*PPR (green) and *La*PPR**^Mu2^** (blue); (b) Ramachandran plot of *La*PPR**^Mu2^** structure. The initial structure of *La*PPR**^Mu2^** was predicted from AlphaFold.


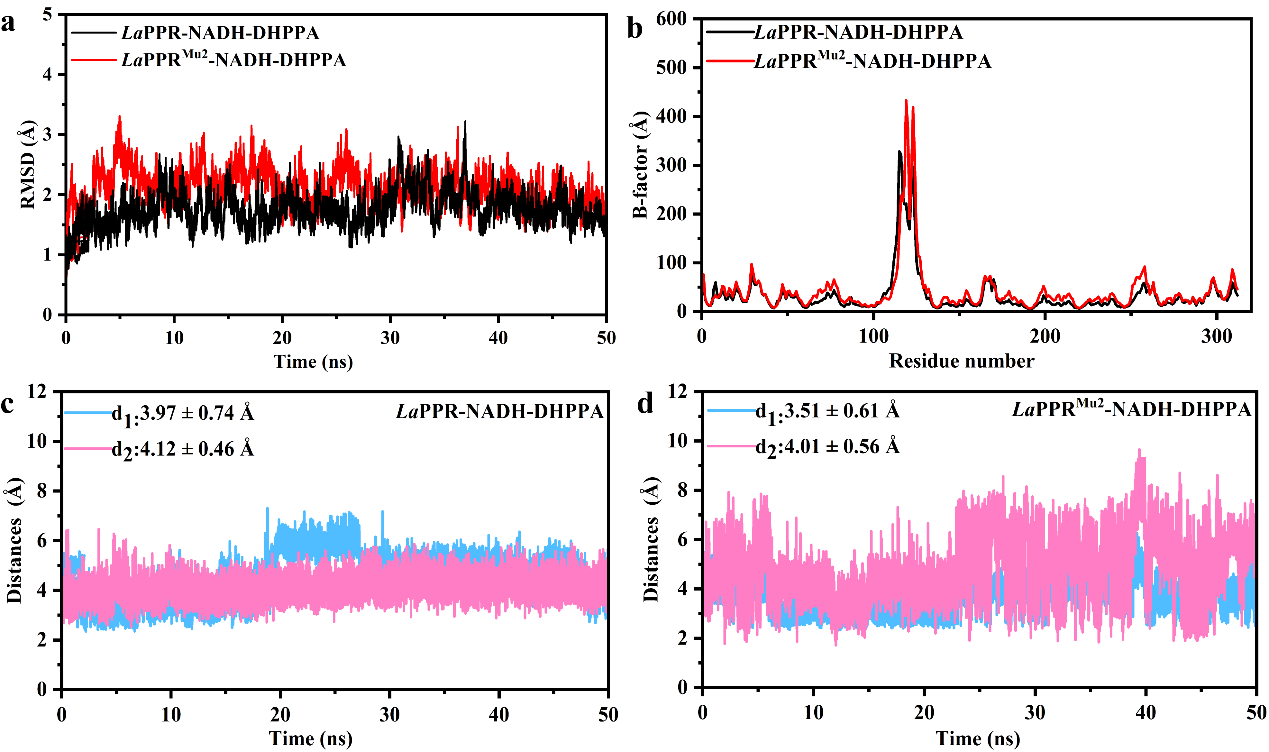
 Additional file 1: Figure **S8.** (a) Root-mean-square-deviation (RMSD) calculated from MD simulations

of *La*PPR-NADH-DHPPA and *La*PPR**^Mu2^**-NADH-DHPPA complex. (b) B-factor calculated from MD simulations of *La*PPR-NADH-DHPPA and *La*PPR**^Mu2^**-NADH-DHPPA complex. (c) Distances of hydride transfer and proton transfer (d_1_ and d_2_) calculated from 50 ns MD simulations on the *La*PPR-NADH-DHPPA complex. (d)Distances of hydride transfer and proton transfer (d_1_ and d_2_) calculated from 50 ns MD simulations on the *La*PPR**^Mu2^**-NADH-DHPPA complex. Mean ± standard deviation is shown for 25000 snapshots of the 50 ns MD simulations. The initial structure of *La*PPR**^Mu2^** was predicted from AlphaFold.

**
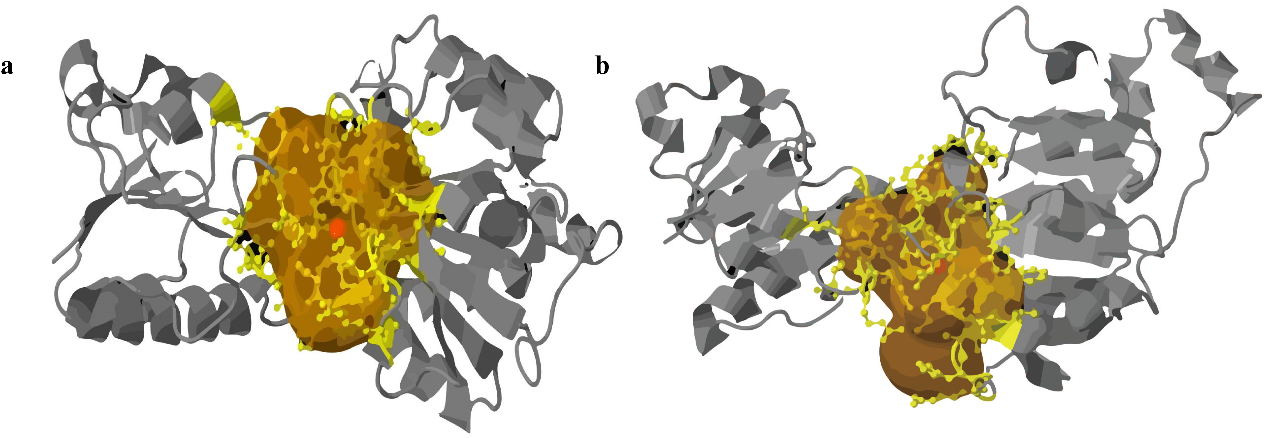
** Additional file 1: **Figure S9.** (a) The wild-type *La*PPR pocket, shown in yellow, has a volume size of 989 Å^3^; (b)The mutant *La*PPR**^Mu2^** pocket, shown in yellow, has a volume size of 1359 Å^3^. The pocket volume for each of the structures has been computed with the POCASA server. The initial structure of *La*PPR**^Mu2^** was predicted from AlphaFold.

**
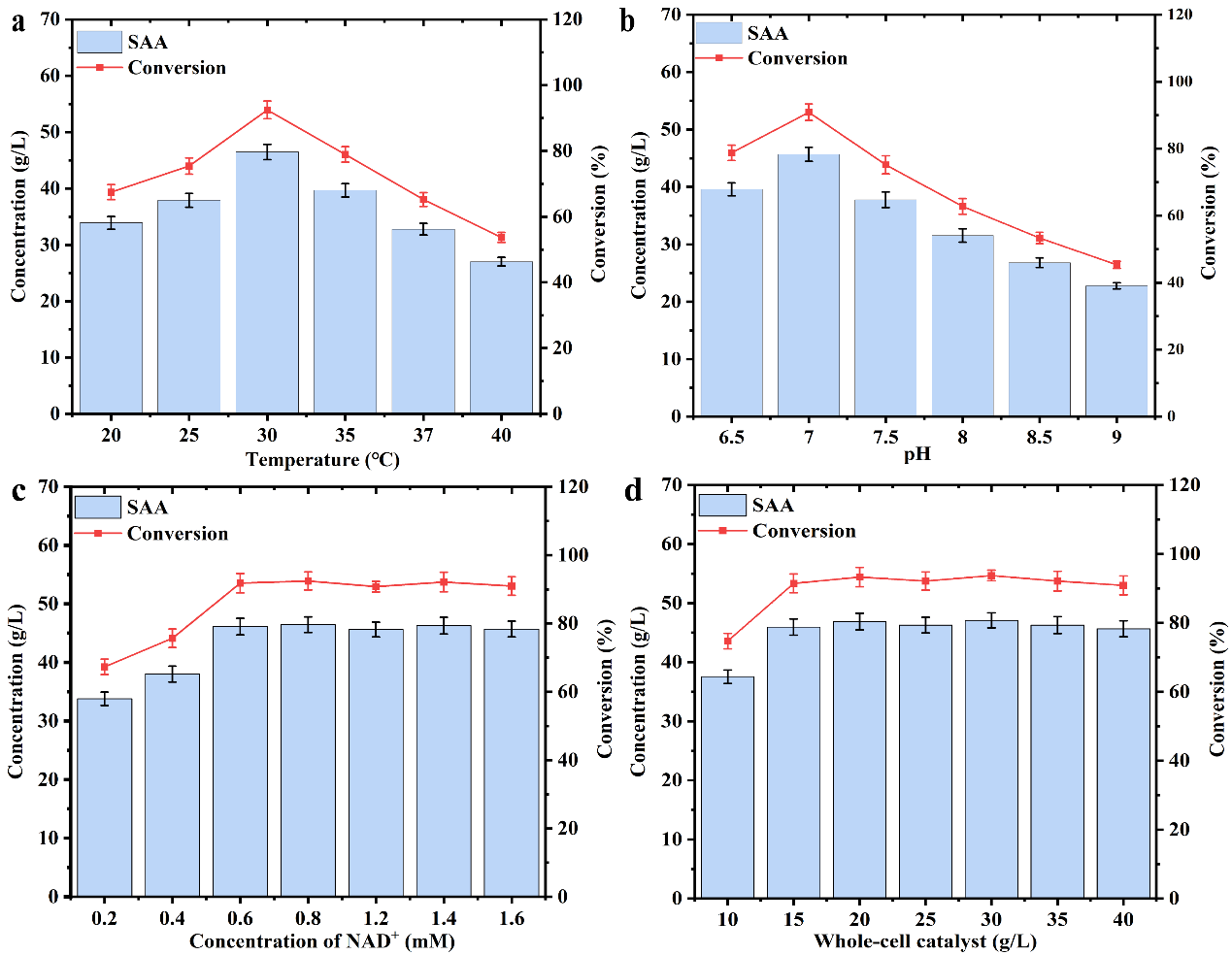
** Additional file 1: **Figure S10.** (a) Effect of conversion temperature on SAA concentration. The conversion reactions were performed in a 10 mL volume of 50 mM Tris-HCl buffer, pH 7.0, containing 3 mM NAD^+^, with 20 g L^-1^ wet whole-cell biocatalysts, 50 g L^-1^ L-DOPA and 60 g L^-1^ sodium formate, in 20-40 °C for 12 h. (b) Effect of conversion pH on SAA concentration. The conversion reactions were performed in a 10 mL volume of 50 mM Tris-HCl buffer, pH 6.5-9.0 containing 3 mM NAD^+^, with 20 g L^-1^ wet whole-cell biocatalysts, 50 g L^-1^ L-DOPA and 60 g L^-1^ sodium formate, in 30 °C for 12 h. (c) Effect of concentration of NAD^+^ on SAA concentration. The conversion reactions were performed in a 10 mL volume of 50 mM Tris-HCl buffer, pH 7.0, containing 0.2-1.6 mM NAD^+^, with 20 g L^-1^ wet whole-cell biocatalysts, 50 g L^-1^ L-DOPA and 60 g L^-1^ sodium formate, in 30 °C for 12 h. (d) Effect of concentration of whole-cell catalyst on SAA concentration. The conversion reactions were performed in a 10 mL volume of 50 mM Tris-HCl buffer, pH 7.0, containing 0.6 mM NAD^+^, with 10-40 g L^-1^ wet whole-cell biocatalysts, 50 g L^-1^ L-DOPA and 60 g L^-1^ sodium formate, in 30 °C for 12 h. The data represent mean ± SD, as determined from three independent experiments.
